# Genome Structure and Reproductive Behaviour Influence the Evolutionary Potential of a Fungal Phytopathogen

**DOI:** 10.1371/journal.ppat.1003020

**Published:** 2012-11-08

**Authors:** Guillaume Daverdin, Thierry Rouxel, Lilian Gout, Jean-Noël Aubertot, Isabelle Fudal, Michel Meyer, Francis Parlange, Julien Carpezat, Marie-Hélène Balesdent

**Affiliations:** 1 INRA, UR 1290 Bioger, Campus AgroParisTech, Thiverval-Grignon, France; 2 AgroParisTech, Campus AgroParisTech, Thiverval-Grignon, France; 3 INRA, UMR 1248 AGIR, Castanet Tolosan, France; 4 CETIOM, Campus AgroParisTech, Thiverval-Grignon, France; University of Melbourne, Australia

## Abstract

Modern agriculture favours the selection and spread of novel plant diseases. Furthermore, crop genetic resistance against pathogens is often rendered ineffective within a few years of its commercial deployment. *Leptosphaeria maculans*, the cause of phoma stem canker of oilseed rape, develops gene-for-gene interactions with its host plant, and has a high evolutionary potential to render ineffective novel sources of resistance in crops. Here, we established a four-year field experiment to monitor the evolution of populations confronted with the newly released *Rlm7* resistance and to investigate the nature of the mutations responsible for virulence against *Rlm7*. A total of 2551 fungal isolates were collected from experimental crops of a *Rlm7* cultivar or a cultivar without *Rlm7*. All isolates were phenotyped for virulence and a subset was genotyped with neutral genetic markers. Virulent isolates were investigated for molecular events at the AvrLm4-7 locus. Whilst virulent isolates were not found in neighbouring crops, their frequency had reached 36% in the experimental field after four years. An extreme diversity of independent molecular events leading to virulence was identified in populations, with large-scale Repeat Induced Point mutations or complete deletion of *AvrLm4-7* being the most frequent. Our data suggest that increased mutability of fungal genes involved in the interactions with plants is directly related to their genomic environment and reproductive system. Thus, rapid allelic diversification of avirulence genes can be generated in *L. maculans* populations in a single field provided that large population sizes and sexual reproduction are favoured by agricultural practices.

## Author Summary

Plant disease resistance often relies on simple “gene-for-gene” systems and, in the pathogen, a mutation in a single “avirulence” gene matching the plant resistance gene is sufficient to render the resistance ineffective. In agricultural systems, breeding for resistance is challenged by both the high evolutionary potential of the pathogen and the large scale of crop production; together, these factors encourage “breakdown” of novel sources of resistance soon after their deployment. Here, we established a four-year field experiment to evaluate the mechanisms and speed with which a fungal pathogen of oilseed rape, *Leptosphaeria maculans* renders ineffective the novel resistance gene *Rlm7*. The pathogen showed a very high evolutionary potential; the proportion of isolates in the population that were virulent against *Rlm7* increased from 0 to 36% in four years. The experiment demonstrated that an extremely diverse range of molecular events leading to virulence, from more or less extensive nucleotide mutations or deletions to complete gene deletion, can occur in a single field. These results suggest that the genomic environment of the avirulence gene and the reproductive regime of the pathogen promote mutability at a single locus to produce virulence. Cropping practices that promote large pathogen populations and encourage sexual reproduction therefore favour rapid adaptation of the pathogen to the novel resistance.

## Introduction

Fungi are the most important pathogens of cultivated plants, with significant economic, food security and environmental impacts, the latter being due to the large quantities of fungicides used to control plant diseases [1]. In contrast to fungicide use, genetic resistance against pathogens in crops is an environmentally friendly strategy to control diseases. Frequently, effective resistance has been provided by the introduction of major resistance (R) genes into crop genotypes [2]. Unfortunately, fungal pathogens have an incredible plasticity with which they can respond to their environment. Furthermore, their ability to adapt to changes in their environment and to disseminate these adaptations makes them very successful in countering crop defenses and control methods [1]. The rapid emergence of new strains able to render ineffective new R genes is thus a common feature of fungal phytopathogens [3].

Durable disease resistance has always been a goal of plant breeding programs since it is cost-effective and environmentally friendly whilst promoting the conservation of rare genetic resources [4]. Durability of resistance is largely dependent on the biology of the pathogen and the evolutionary potential of the pathogen population [3]. In addition, cropping practices such as crop rotation and stubble management may directly affect the evolutionary potential of the pathogen by reducing its population size or dispersal or by interfering with its reproductive regime [5].

Major gene resistance depends on the “gene-for-gene” concept in which the host R protein interacts with a corresponding pathogen avirulence (Avr) protein to initiate plant disease responses and resistance [6]. Avr proteins are now known to be effectors involved in plant pathogenesis that were recognised by the plant surveillance machinery in the course of plant-pathogen co-evolution [7]. Thus, avoidance of recognition by host R proteins often involves pathogen effector gene inactivation, which may result in a fitness penalty for the pathogen [4]. An additional level of complexity arises because many fungal or oomycete pathogens have their effector genes in non-conventional, adaptable, regions of their genomes [8]. It was recently suggested that the location of effector genes of *Leptosphaeria maculans*, the cause of “phoma stem canker of oilseed rape”, in AT-rich, transposable element (TE)-rich blocks of the genome has major implications for gene mutability, resulting in either allelic diversification or gene inactivation under *R* gene selection [9]. For example, it was suggested that Repeat Induced Point mutation (RIP), a fungal-specific mechanism of inactivation of repeated sequences in genomes, was acting on single-copy genes embedded in TE-rich blocks of the genome to favour the allelic diversification of such genes [9], [10], [11].

The cloning and characterization of the molecular determinants of resistance from the plants (*R* genes) and pathogens (*Avr* genes) has not only improved understanding of their functions and interactions but also provided the molecular information for detecting mutations in these genes. Fungal *Avr* genes have been cloned from only seven species, and the mechanisms by which fungal *Avr* genes evolve to evade host recognition is only documented for few *Avr* genes, mostly in *Cladosporium fulvum*, *Magnaporthe oryzae*, *Rhynchosporium secalis* and *L. maculans* [12], [13]. Moreover the link between laboratory and field studies has often been missing, and when field populations of pathogens were investigated for mutations in *Avr* genes they have often been isolated from uncharacterized crop genotypes or have been collected and analysed a long time after the corresponding *R* gene had been commercially deployed in crops (e.g. [14]). There is currently no example of identification of the initial events leading to virulence when a fungal pathogen is exposed for the first time to an *R* gene deployed under field conditions.

*L. maculans* is a pathogen with a high evolutionary potential combining large population size, mixed reproduction regime and high dispersal ability. Following sexual reproduction taking place on stem debris, leaf infections by ascospores (in autumn in western Europe) cause phoma leaf spots, supporting asexual multiplication. The life cycle of the pathogen is completed by a lengthy symptomless colonisation phase when the pathogen grows from the leaf lesions along the petiole to the stem, where cankers develop to cause lodging and yield losses in crops at the end of the growing season (spring and early summer in western Europe). Sexual mating between the numerous isolates that colonised the stem tissues then takes place [12]. Major gene resistance against *L. maculans* has been widely used in oilseed rape [15], [16], but was rendered ineffective in only a few growing seasons [17], [18].

Three *L. maculans* Avr genes, *AvrLm1*, *AvrLm6* and *AvrLm4-7* have been cloned [19]–[21]. All encode Small Secreted Proteins (SSPs) embedded in large TE-rich blocks of the genome termed AT-isochores. *AvrLm4-7* is located within a 96-kb AT-isochore which only contains two other genes located 26 and 28 kb away. These two genes are also predicted to encode SSPs. *AvrLm4-7* is recognised by two distinct *R* genes, *Rlm4* and *Rlm7* and escape from recognition by *Rlm4* is due to a single-base non-synonymous mutation resulting in a Gly120Arg change in the protein. This change does not alter recognition by *Rlm7* [21]. Before 2003, *L. maculans* has not been exposed to the *Rlm7* selection in Europe and a population survey done in 2000–2001 in France identified only one virulent isolate out of 1787 isolates (0.05%) [22]. *Rlm7* has been introduced in commercial cultivars in 2003 in France, with only 1% of the hectarages cropped with *Rlm7* cultivars until 2005 (X. Pinochet, CETIOM, personal communication), thus providing us with the opportunity to survey emergence of virulent *L. maculans* isolates at the time of initial selection pressure and to identify the first molecular events responsible for the overwhelming of the resistance gene.

Here, we established a four-year field experiment (Figure S1 in Text S1, Figure S2 in Text S1) and combined molecular genetic and population genetic approaches to evaluate speed and patterns of mutations in the *AvrLm4-7* gene responsible for the loss of the *AvrLm7* specificity in *L. maculans* populations exposed to *Rlm7* selection. Molecular analysis of events leading to the virulent phenotype in a single field revealed a tremendous diversity of mutation events, and confirmed the importance of the genomic environment in gene mutability.

## Results

### Changes in the frequency of virulent isolates in the experimental field

**Figure 1:**
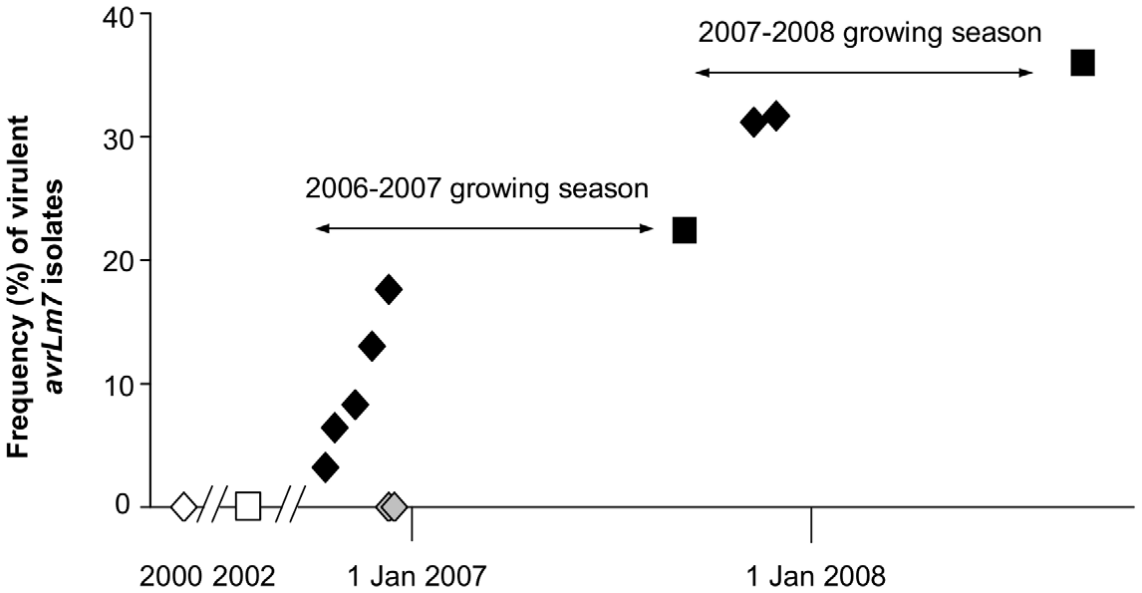
Changes in the frequency of virulent *avrLm7* isolates of *Leptosphaeria maculans* obtained from susceptible cultivars. A total of 1278 isolates were recovered from susceptible cultivars (without the resistance gene *Rlm7*) sown next to resistant (with *Rlm7*) cultivars in the experimental plot at Grignon and its immediate surroundings between 2000 and 2008 and phenotyped for their virulence towards *Rlm7* cultivars. Isolates were obtained either at the phoma leaf spot stage in autumn (diamonds; beginning of the disease cycle) or at the phoma stem canker stage at harvest (squares, end of the disease cycle, after one sexual reproduction cycle). Isolates were collected before the start of the experiment (white symbols), within the field experiment (black symbols) or in fields sown close (<600 m) to the field experiment (grey symbols).

We established a four-year (2004–2005 to 2007–2008 growing seasons) field experiment at Grignon, France, during which *Rlm7* and *rlm7* cultivars were grown alongside each other (with relative ca. 2/3 of the area cropped with the *Rlm7* cultivar and 1/3 cropped with the *rlm7* cultivar) (Figure S1 in Text S1, Figure S2 in Text S1). Exposure of the *L. maculans* population to *Rlm7* was maximised because there was no crop rotation or ploughing in crop debris. Before the start of the experiment at Grignon (i.e. between 2000 and 2004) no *Rlm7* cv. had been grown and the frequency of virulent *avrLm7* isolates was minimal at both Grignon and another crop located 12 km away at Versailles (Figure S1A in Text S1), with estimated frequencies of *avrLm7* isolates ranging from 0.006% to 1.3% at Versailles and from 0 to 1.3% at Grignon (Figure 1, Table 1, Table S1 in Text S1).

**Table 1:** Changes in frequency of virulent *avrLm7* isolates in *Leptosphaeria maculans* populations before and during exposure to the *Rlm7* selection in a field experiment at Grignon and control plots at Grignon and Versailles.

	Frequency (%) of virulent isolates (number of isolates tested) in			
	Versailles	Grignon		
Year^a^	*rlm7* b plants	Field experiment on *rlm7* plants	Field experiment on *Rlm7* plants	Control plots (<600 m) on *rlm7* plants
2000	0% [0, 4.1]^c^ (87)	0% [0, 3.6] (100)	na^d^	nd
2002	0% [0, 3.8] (95)	0% [0, 1.9] (191)	na	nd
2003	2.2% [0.05, 11.5] (46)	nd	na	nd
2004	0% [0, 1.7] (210)	nd	na	nd
2006	0% [0, 1.5] (246)	9.3% [6.7, 12.3] (445)	40.0% [23.9, 57.9] (35)	0% [0, 3.9] (93)
2007	0.7% [0, 3.4] (160)	31.5% [24.7, 38.8] (178)	94.2% [89.7, 97.0] (187)	nd
2008	nd	36.2% [30.0, 42.7] (235)	94.6% [91.8, 96.7] (355)	nd

a The field experiment was established in autumn 2004 and the first samples from it were in autumn 2006.

b *rlm7* (without the *Rlm7* gene) plants were sown and used as trap cultivars to estimate the proportion of virulent isolates present in the air and landing on the crop at each site in each year.

c Values in square brackets represent the exact confidence interval of the percentages at a 95% confidence level based on the total number of isolates and the percentage of virulent isolates in the sampling; values in brackets indicate the total number of isolates analysed for each sampling.

d na, not applicable (the resistance gene was not yet released); nd, not determined.

In the experimental field, the phoma stem canker was not severe in the summer of 2007 or 2008. The G2 disease indices, a disease severity index summarising the proportions of plants observed within six canker severity classes and ranging between 0 (all plants healthy) to 9 (all plants with severe canker) [4], were 1.53 (*rlm7* cv.) and 0.86 (*Rlm7* cv.) in 2007. However, the G2 index had increased to 2.34 on the *Rlm7* cultivar by 2008, reflecting a localized overwhelming of the *Rlm7* resistance during the course of the experiment.

In the course of the four-year experiment, we collected 2551 isolates either from the *Rlm7* cv. Exagone or from the *rlm7* genotype Campala (Grignon and Versailles; Table S2 in Text S1). Of these, 1987 isolates were characterized for their interactions with *Rlm4* and *Rlm7* plant genotypes (Table S2 in Text S1). The number of virulent *avrLm7* isolates remained low or undetectable in the control plot at Versailles on the susceptible cv. Campala. At Grignon, the frequency of virulent *avrLm7* isolates on the susceptible *rlm7* cv. grown next to the *Rlm7* cultivar steadily increased to 36.2% of the population, whilst *avrLm7* isolates were not detected in crops located less than 600 m from the experimental plot (Figure 1, Table 1, Figure S1 in Text S1, Table S2 in Text S1). Consistent with analyses of *L. maculans* ascospore dispersal indicating that most spores are deposited within 500 meters from the source and especially in the first 100 meters [23], [24], this suggests that the observed increase in *avrLm7* isolate frequency in the field experiment was due to the recurrent local use of *Rlm7* and not to an increase of its frequency at the regional level.

### *AvrLm4-7* polymorphism in avirulent population

**Figure 2:**
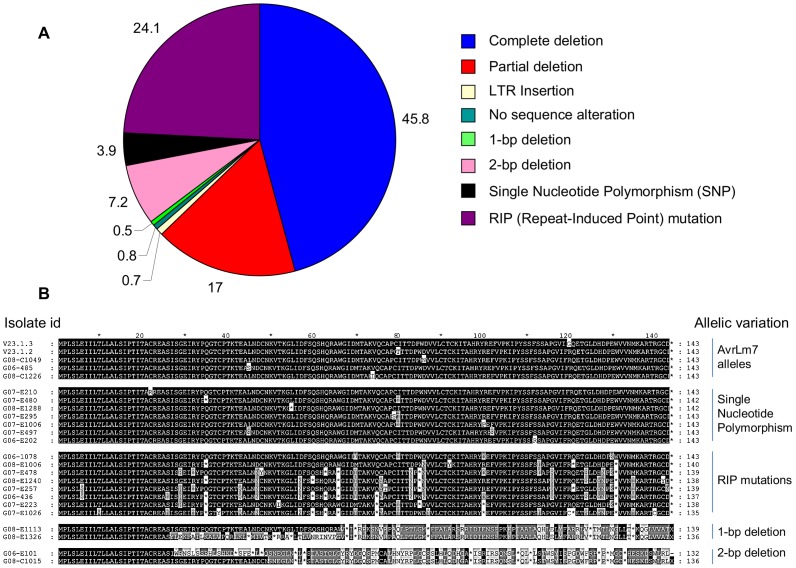
Main mutational events identified in the *avrLm7* alleles and consequences for the protein produced. **A.** Percentages of the main mutational events in the collection of 769 virulent *avrLm7* isolates analysed (excluding nine isolates for which the mutation events could not be characterized). All isolates were obtained from the experimental plot at Grignon between 2006 and 2008. **B.** GeneDoc-edited alignment of representative variants of the AvrLm4-7 protein. The AvrLm4-7 protein sequences deduced for three avirulent isolates with the *AvrLm7* specificity, and 19 virulent *avrLm7* isolates with different mutation events leading to virulence are shown and compared to those of the two reference isolates v23.1.3 and v23.1.2. The intensity of shading reflects the intensity of sequence conservation. Stop codons are indicated as stars.

Nucleotide polymorphisms in *AvrLm4-7* were analysed in 169 *AvrLm7* isolates from the sample. A very low level of sequence polymorphism was found within the gene with only five polymorphic nucleotides, all corresponding to non-synonymous mutations in the protein (Table 2, Figure 2B). The combination of these polymorphic sites generated five haplotypes (Table 2). In accordance with previous data [21], one haplotype included those of the isolates that showed the AvrLm4 specificity and differed from the other haplotypes by the presence of a guanine at base 358 resulting in a glycine at amino acid 120 (Table 2). All other mutations were found in isolates that had lost the *AvrLm4* specificity but maintained the *AvrLm7* avirulence, thus defining four distinct alleles for *avrLm4-AvrLm7* isolates (Table 2).

**Table 2:** Amino acid polymorphisms found in AvrLm4-7 proteins produced by different alleles of the avirulent *AvrLm7* form of the gene and their occurrence in populations sampled from the field experiment at Grignon.

Interaction phenotype alleles^a^	Amino acid position^b^					No (frequency)^c^ of isolates
	45	74	80	86	120	
*AvrLm4-AvrLm7*	Leu	Val	Ile	Asp	Gly	7 (4.1%)
*avrLm4-AvrLm7*	Leu	Val	Thr	Asp	Arg	62 (36.7%)
*avrLm4-AvrLm7*	Leu	Val	Ile	Asn	Arg	86 (50.9%)
*avrLm4-AvrLm7*	Ser	Val	Ile	Asp	Arg	13 (7.7%)
*avrLm4-AvrLm7*	Leu	Ile	Ile	Asp	Arg	1 (0.6%)

a *avrLm4-AvrLm7*, isolates avirulent on *Rlm7* only; *AvrLm4-AvrLm7*, isolates avirulent on both *Rlm4* and *Rlm7*.

b Comparisons are based on the sequence of the reference AvrLm4-7 protein of isolate v23.1.3 inducing avirulence towards *Rlm4* and *Rlm7* genotypes. Leu45Ser is due to a T to C mutation at base 134; Val74Ile is due to G to A mutation at base 220; Ile80Thr is due to a T to C mutation at base 239; Asp86Asn is due to a G to A mutation at base 256; Gly120Arg is due to a G to C mutation at base 358. The underlined amino acids are those showing polymorphism as compared to the reference sequence.

c Number (frequency) of *AvrLm7* isolates showing a given combination of amino acid substitution at positions 45, 74, 80, 86 and/or 120.

### An extremely diverse range of mutational events is responsible for virulence against *Rlm7*

Of the 808 virulent *avrLm7* isolates obtained from Grignon, 769 were characterized for molecular events responsible for virulence. Numerous mutational events responsible for loss of the avirulence function were identified, single-or di-nucleotide deletions, single nucleotide polymorphisms (SNPs), wide degeneracy due to RIP, complete or partial deletion of the gene, major chromosomal rearrangements and alteration in gene expression. Only a few isolates had SNPs, single-nucleotide deletions, or under-expression of the gene whereas gene deletions and RIP mutations were present in 62.8% and 24.1% of the virulent isolates, respectively (Figure 2A). In most cases the mutational events resulted in lack of protein production or production of a severely truncated protein, while in a few other cases the 3-D structure of the protein was probably modified (e.g. mutation in cysteine residues involved in disulfide bridge formation) (Figure 2B, Table 3). Lastly, in a few cases, the sequence of the gene was unaltered but its expression *in planta* was impaired (Figure S3 in Text S1) suggesting mutation of regulatory elements outside of the gene sequence.

**Table 3:** Point mutations in the *AvrLm4-7* gene linked to the virulent *avrLm7* phenotype found in the Grignon populations during the experiment.

Nucleic acid change			Amino acid change			No of isolates^a^	Representative isolate
T^64^	→	C^64^	Cys^22^	→	Arg^22^	1	G07-E210
C^103^	→	T^103^	Gln^35^	→	STOP	7 (6)	G07-E480
T^164^	→	A^164^	Leu^55^	→	STOP	2	G08-E1288
G^236^	→	C^236^	Cys^79^	→	Ser^79^	1	G07-E295
G^299^	→	C^299^	Arg^100^	→	Pro^100^	16 (9)	G07-E1006
T^305^	→	C^305^	Phe^102^	→	Ser^102^	1	G07-E497
C^336^	→	G^336^	Ser^112^	→	Arg^112^	2	G06-E202

a In parentheses, number of isolates when excluding isolates obtained from the same stubble, which may represent sister isolates from the same cross.

#### Point mutations and other minor events

Of the 769 isolates analysed, 287 isolates (37.3%) produced an *AvrLm4-7* amplicon of the expected size. Sequencing of the PCR products revealed four discrete mutational events, point deletions, dinucleotide deletions, point mutations not attributable to RIP and RIP mutations (Figure 2B).

Single or di-nucleotide deletions introduced a frameshift likely to result in production of truncated proteins sized 20 to 46 amino acids (Figure 2B). Single nucleotide deletion was a rare event that occurred at only two sites in four isolates. A more common event, found in 55 isolates (7.1% of the *avrLm7* isolates analysed) was the deletion of an AA dinucleotide from one of two locations within the coding sequence: AA78–79 (53 isolates) and AA149–150 (2 isolates).

Seven distinct SNPs were found in 30 virulent isolates (Figure 2, Table 3). All mutations were either non-sense or missense mutations and they resulted in introduction of stop codons or drastic changes in the physico-chemical properties of the residues (Figure 2, Table 3).

#### RIP mutations

**Figure 3:**
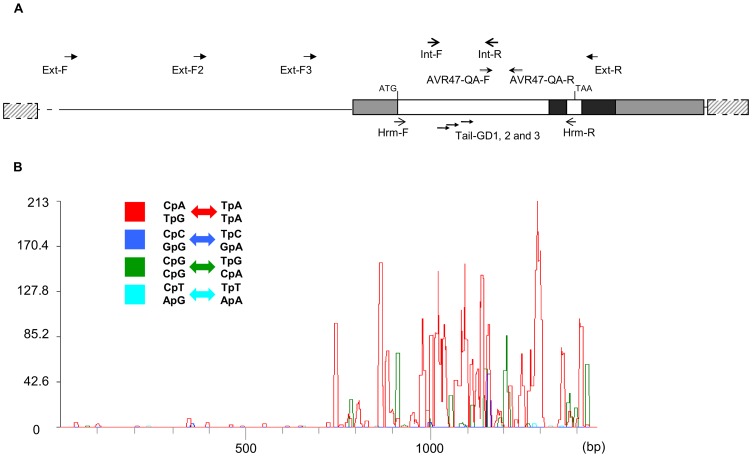
Schematic representation of the *AvrLm4-7* gene and RIPCAL analysis of 127 RIPped *AvrLm4-7* alleles. **A.** The *AvrLm4-7* sequence analysed (encompassing ca. 550 bp of the promoter region and ca. 60 bp of the 3′UTR) and the location of primers used for PCR and sequencing (primers Ext-F, Ext-R, Ext-F2 and Ext-F3, Int-F and Int-R), qRT-PCR (AVR47-QA-F and AVR47-QA-R), TAIL-PCR (Tail-GD1-Tail-GD3) and HRM analyses (Hrm-F and Hrm-R) are shown. White boxes, coding sequence; black boxes, introns; grey boxes, 5′ and 3′ UTR; hashed boxes, transposable elements. **B.** The alleles were identified in virulent *avrLm7* isolates obtained in 2006 to 2008 from the experimental field at Grignon. The sequence with the greatest total G+C content (v23.1.2) was chosen as the least RIP-mutated model for comparison of all aligned sequences. Physical distribution of RIP along the *AvrLm4-7* gene is illustrated by the overall RIP mutation frequency over a 50 bp scanning window. A substantial over-representation of the CpA/TpG↔TpA mutation (red) over CpN↔TpN or NpG↔NpA mutations (blue and green curves) was observed.

There were 183 isolates with multiple point mutations (three to 31; 23.8% of the virulent isolates analysed) that corresponded to 84 polymorphic sites throughout the *AvrLm4-7* sequence. Alignment of 127 fully sequenced alleles showed that these mutations were exclusively C to T or G to A mutations. This resulted in a reduced GC content of the alleles (mean: 40.45% instead of 43.93% in the reference v23.1.3 allele). The mutations were not random and preferentially affected CpA or TpG di-nucleotides (60.7% of the cases), the typical dinucleotide context for RIP [25] (Figure 3). Fifty-one out of 56 favoured RIP targets were mutated in one or other allele. In these isolates, the whole gene sequence and the 5′ and 3′ UTR were severely affected by RIP, except for the intron and part of the sequence coding for the signal peptide, which is consistent with the scarcity of RIP targets in these parts of the gene (Figure 3). In contrast, the 128-bp sequenced part of the promoter upstream of the 5′ UTR was not affected by RIP (Figure 3). RIP mutations often introduced stop codons in the sequence (Figure 2B) and generated a high allelic diversity, with 98 distinct alleles of which only seven were common to more than one isolate (2 to 4).

One additional level of complexity was observed in 49 isolates for which unresolved bases (reproducible presence of a double A/G or C/T peak in the chromatograms) were present at some sites in the sequence of the RIPped alleles.

**Figure 4:**
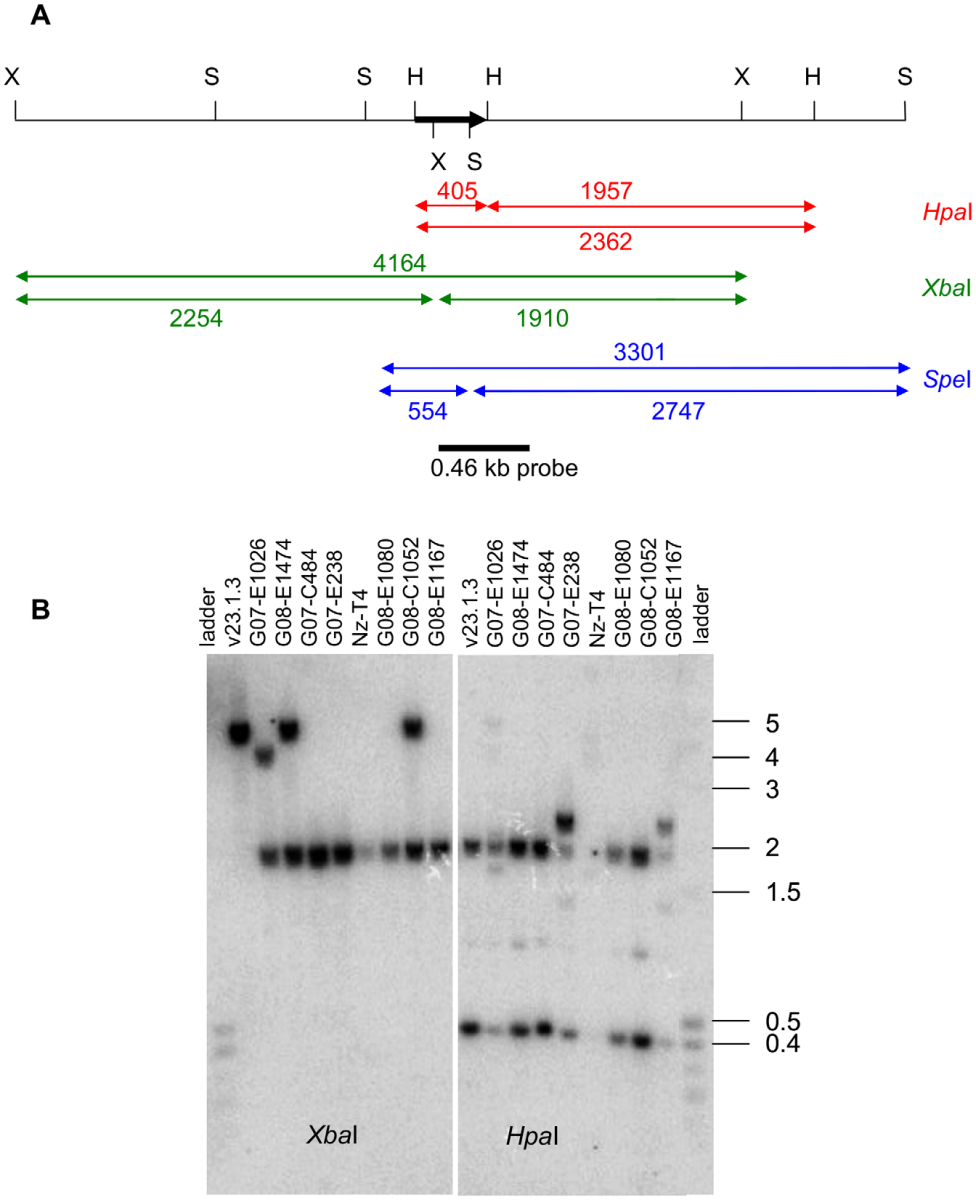
Southern-blot analysis of RIPped and duplicated alleles of *AvrLm4-7* in virulent isolates. **A.** schematic representation of the 4.5-kb region containing the *AvrLm4-7* gene. The restriction sites above the line are present in the control v23.1.3 isolate and are indicated as follows: *Xba*I, X; *Hpa*I, H; and *Spe*I, S. The *Xba*I and *Spe*I sites indicated below the *AvrLm4-7* gene are absent in v23.1.3 and are generated by RIP mutations in some RIPped alleles (see also Table S3 in Text S1). The thick black arrow indicates the *AvrLm4-7* gene and its direction of transcription. The location of the hybridization probe is indicated by the thick black bar. Red, green and blue lines indicate the location and size (in bp) of the restriction fragments generated by *Hpa*I, *Xba*I and *Spe*I, respectively, in the avirulent isolate v23.1.3 (upper double arrow(s)) and in representative RIPped isolates (lower double arrow(s)). **B.** Southern blot of the reference isolates v23.1.3 (*AvrLm7*) and Nz-T4 (*avrLm7*), along with *avrLm7* field isolates with different mutational events leading to virulence; G07-E1026, G07-E238, and G08-E1080, RIPped alleles; G08-E1474, G07-C484, and G08-C1052, RIPped alleles with duplicated copies of the gene, G08-E1167, lack of PCR amplification (for *Spe*I data, see Table S3 in Text S1). Size of the GeneRuler 1 kb Plus DNA ladder (in kb) is indicated on the right.

Southern blot analysis was done for two reference isolates [v23.1.3 (*AvrLm7*) and Nz-T4 (*avrLm7*)] and for 17 of the virulent *avrLm7* isolates collected. The probe used was a 0.46-kb portion of the *AvrLm4-7* gene (Figure 4A) and included one *Hpa*I and no *Xba*I nor *Spe*I restriction sites. These restriction enzymes were used to digest genomic DNA. For all isolates with RIPped alleles, the hybridization pattern differed from that of the avirulent v23.1.3 isolate for at least one of the restriction enzymes used (Figure 4B, Table S3 in Text S1). These patterns were fully explained by the *AvrLm4-7* sequence polymorphism in these isolates, with RIP mutations modifying the restriction sites. For example, RIP mutations in the *AvrLm4-7* gene of isolate G08-E1080 resulted in the generation of a G→A polymorphism at base 85, generating a TCTAGA *Xba*I restriction site and resulting in the restriction of the 4.2 kb fragment into two fragments sized 2.3 kb and 1.9 kb, with only the shorter fragment being detected by the probe (Figure 4). In five isolates (G07-E1026, G08-E1474, G08-C1052, G07-E238, G07-C484), however, an additional fragment was detected for at least one restriction enzyme (Figure 4B, Table S3 in Text S1) suggesting that they had two copies of the gene. For instance, *Xba*I restriction patterns obtained for isolates G08-C1052 and G08-E1474 revealed two bands, the 4.2 kb band corresponding to that of v23.1.3 and the 1.9 kb band corresponding to the fragment generated by the RIP mutation at base 85. For the G07-E1026 isolate, a different *Xba*I position at the left border of the flanking genomic region of the *avrLm4-7* gene could explain the hybridization of a smaller-than-expected fragment (4.0 kb instead of 4.2 kb). Two other virulent isolates showed the presence of a polymorphism at an *Hpa*I restriction site (G/ATTAAC) following RIP mutations. This resulted in absence of the *Hpa*I site and the lack of restriction of a 2.4 kb band into the 0.4 kb and 1.9 kb fragments (Figure 4). Among these five isolates, three belonged to the group of 49 isolates with a few unresolved bases in the sequence of *AvrLm4-7*, but the two others showed unambiguous chromatograms for this sequence. Because (i) most isolates analysed by Southern blot were single-ascospore isolates, (ii) the occurrence of a few bases with double peaks in sequence chromatograms of their *AvrLm4-7* gene was reproducibly observed with different DNA extractions or PCR amplifications and (iii) the clonality of these isolates was confirmed following amplification of their mating type and minisatellite loci, we concluded from these Southern blot analyses that some isolates may have (at least) two distinct and differentially RIPped copies of *AvrLm4-7*. This is most likely the case for the 49 isolates for which unresolved bases were present at some sites in the sequence of *AvrLm4-7*, but since allele duplication was also observed in two isolates for which well-resolved RIPped sequences were obtained, it is postulated that duplications occurred in more than 6.4% of virulent isolates (i.e. for more than 49 out of 769 genotyped isolates), corresponding to more than 26.8% of the RIPped alleles (49 out of 183).

#### Deletion events and major genomic rearrangements in *avrLm7* isolates

A lack of PCR amplification of the *AvrLm4-7* gene with external and internal primers (Figure 3A) was observed in 348 isolates (45.2%). The complete absence of amplification mostly corresponded to complete deletion of the gene as confirmed by Southern blot analysis (Figure 4B, Table S3 in Text S1). A faint signal was observed for two isolates for which no PCR product was obtained, Nz-T4 and G08-E1167, suggesting that an extremely large number of RIP mutations in the sequence of their *avrLm4-7* allele prevented the hybridization of PCR primers but not that of the probe (Figure 4B). A total of 129 isolates (16.8%) produced an amplification product of the expected size with internal primers but no amplification with external primers and five isolates showed an internal amplification product of the expected size whereas the PCR product obtained with external primers was 270-bp larger than expected.

TAIL-PCR analysis of two isolates for which only part of the gene was amplified indicated the presence of a portion of RIP-inactivated TEs (RLC_Pholy or DTM_Sahanna) [9] in the 3′ part of the gene. For the five isolates with a larger-than-expected amplification product, the 271-bp Long Terminal Repeat (LTR) of RLC-Pholy was inserted 52 bp before the ATG. In the absence of TEs having maintained their transposition activity in the genome [9], all these data suggest that major genome rearrangements are the cause of *AvrLm4-7* inactivation in alleles for which only the internal fragment of the gene could be amplified.

#### Unaltered gene sequences

In six *avrLm7* isolates, sequencing of the *AvrLm4-7* gene encompassing 550-bp of promoter region and 60 bp of 3′UTR did not reveal any nucleotide polymorphism as compared to avirulent *AvrLm7* alleles. A reduced level of *AvrLm4-7* expression *in planta* 7 dpi was observed following qRT-PCR analysis (Figure S3 in Text S1), which could explain the *avrLm7* phenotype in these isolates.

### Dynamics of mutation event frequencies during the three-year survey

**Figure 5:**
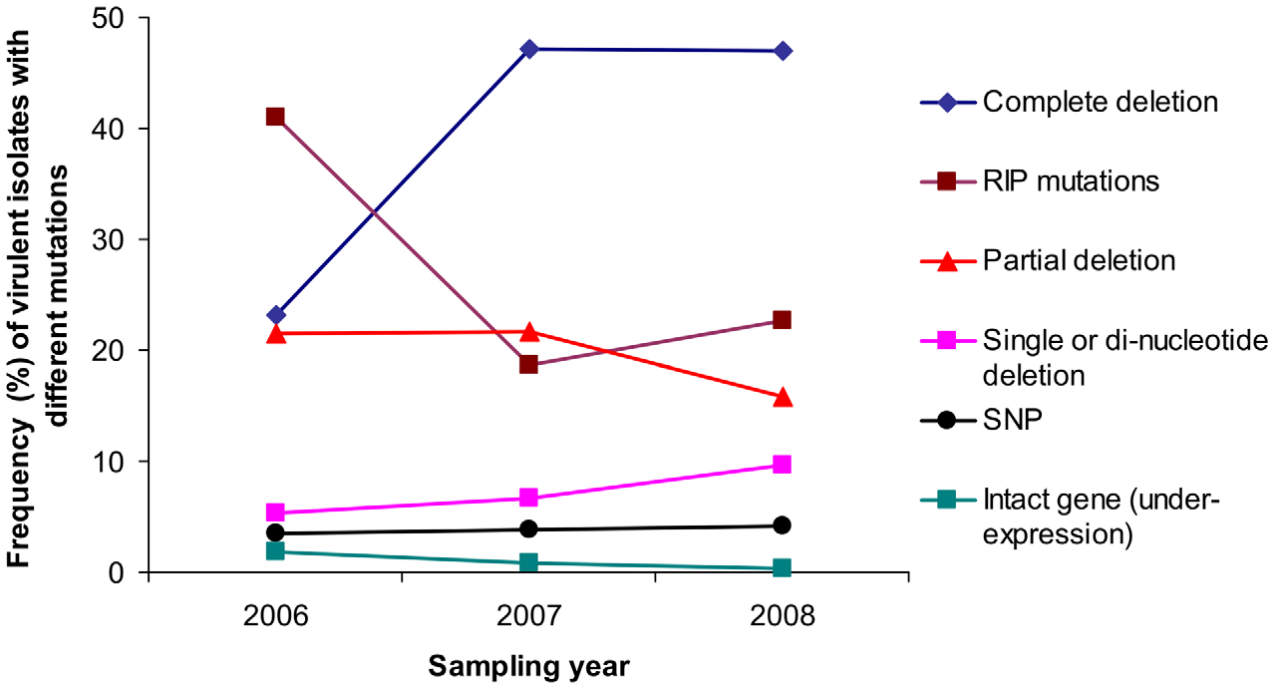
Changes in frequencies of isolates with different mutations leading to the *avrLm7* phenotype over three years in the experimental plot at Grignon. Dark blue diamonds, complete deletions; purple squares, RIP mutations; red triangles, partial deletions; pink squares, AA dinucleotide deletions and point deletions; black dots, Single Nucleotide Polymorphisms; green squares, unaltered gene sequence. In 2007 and 2008, isolates collected the same year at the phoma leaf spot stage and at the stem canker stage from the previous growing season residues have been pooled because they correspond to the same generation after the start of the experiment. In 2006, isolates were collected from phoma leaf spots only.

**Figure 6:**
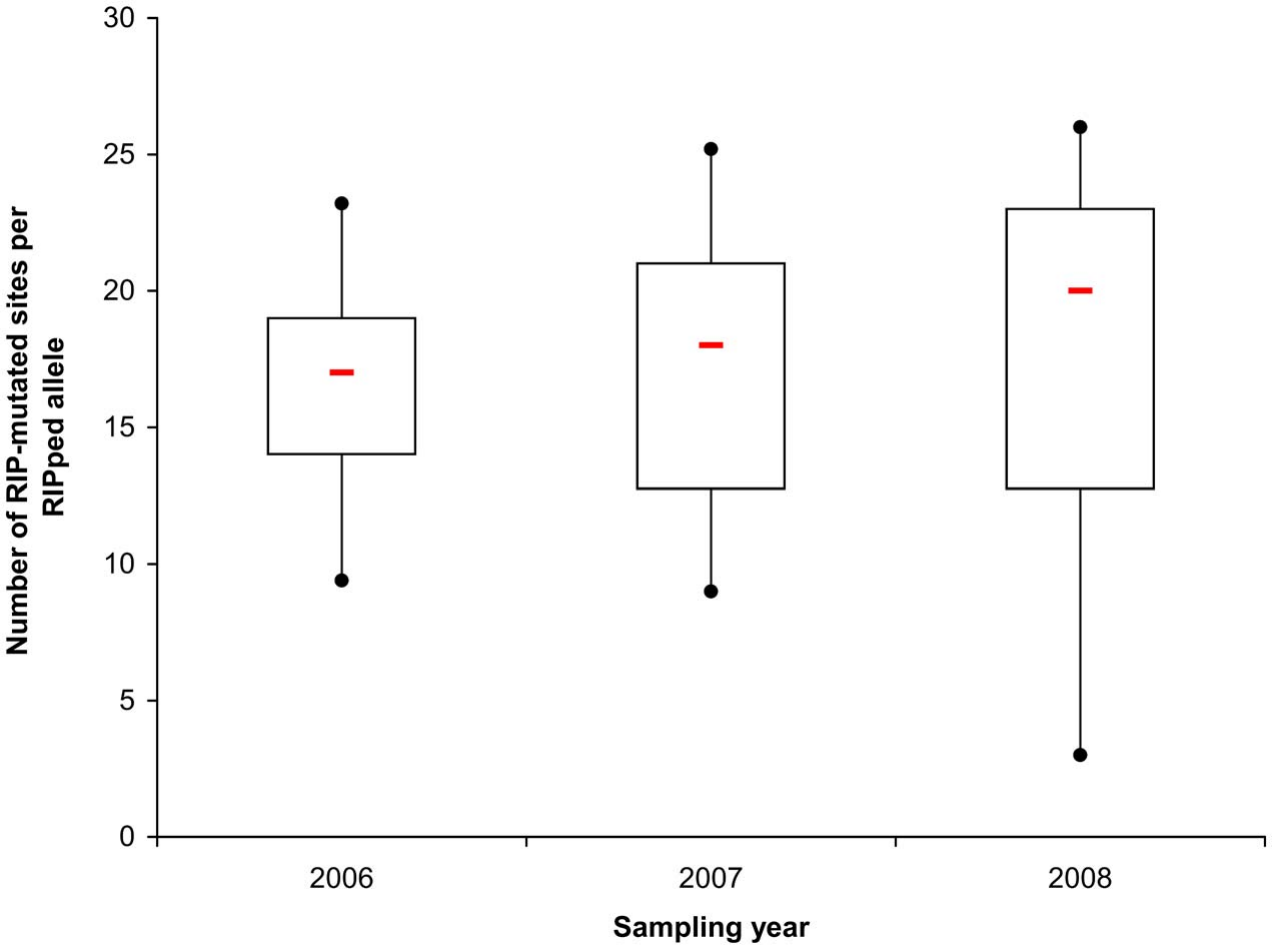
Distribution of the number of RIP mutations in the *AvrLm4-7* gene over sampling years. The number of RIP mutations along the *AvrLm4-7* gene were counted for each RIPped allele and over the three *L. maculans* sexual cycles monitored in the experimental trial. The red line represents the median value; the boxes include values between the first and the third quartile of the distribution; circles, 1st and 9th deciles, respectively. In 2007 and 2008, isolates collected the same year at the phoma leaf spot stage and at the stem canker stage from the previous growing season residues have been pooled because they correspond to the same generation after the start of the experiment. In 2006, isolates were collected from phoma leaf spots only.

In 2000, one *avrLm7* isolate was collected during a large-scale survey of French *L. maculans* populations [22]. In this isolate, the virulent phenotype was due to the insertion of a complete LTR of RLC-Pholy at base 6 of the coding sequence, resulting in the production of a 30 amino acid protein mainly corresponding to part of the TE (data not shown). This event was not identified in the 2006–2008 sampling. Major genome rearrangements leading to complete or partial gene deletion and RIP mutations were the two main events responsible for virulence toward *Rlm7*. However, our survey indicated a contrasting sequence of events (Figure 5). The proportion of the population affected by minor events (SNP, single or di-nucleotide frequency, unaltered gene sequence), or major rearrangements leaving internal part of the gene unaltered remained stable over the three years (Figure 5). In contrast, a significant change in frequency was observed for the two most common events (Pearson's approximate χ^2^ test, with 20,000 random samplings, *P* = 0.022). Whereas RIP mutation was the most frequent event leading to virulence in the first year of the survey, at a time when only very few virulent isolates could be found (Table S2 in Text S1), complete gene deletion became more common in years 2 and 3, while the frequency of RIP mutations decreased in the second year and then remained stable at ca. 20% (Figure 5). When analysing the intensity of RIP mutations, there was no significant increase in the mean number of mutated sites per isolate as a function of the year of isolation (Kruskal-Wallis test; *P* = 0.371, Figure 6) with for example, the same proportion of alleles with the least number of RIP mutations found in autumn 2006 as in summer 2008 (Figure 2, Figure 6), or in contrast, alleles with the greatest number of RIP mutations found in autumn 2006 (isolate G06-436 in Figure 2B).

### Population analysis

Effective population size was firstly estimated using Approximate Bayesian Computation (ABC) methods from the minisatellite polymorphism data obtained for the population sampled in Grignon on the susceptible cv. Campala. Effective population size (*Ne*) estimated at the field scale was 11,500 (CI95% 3,490–29,900).

To investigate the origin of *avrLm7* isolates and how their rapid increase in frequency influenced the genetic structure of *L. maculans* populations, a subset of 161 *avrLm7* and 161 *AvrLm7* isolates collected from the field experiment were genotyped using seven minisatellite (MS) markers located on different chromosomes. All seven markers were polymorphic and a total of 80 alleles were found in the collection. The average number of alleles over loci ranged between 6.86 and 8.86 (Table 4) and revealed no significant difference in allelic variability between virulent and avirulent isolates sampled (Kruskal-Wallis test; *P* = 0.95). Over the three years, the average gene diversity was similar amongst avirulent and virulent isolates (two-sided permutation test, 15,000 permutations, *P* = 0.39). The *AvrLm7* and *avrLm7* isolates collected between autumn 2006 and summer 2008 showed very considerable genotypic diversity and the number of genotypes was identical or very close to the number of isolates (Table 4). Overall, a total of 313 multilocus genotypes (MLG) were differentiated, of which six were shared by two to four isolates and 307 isolates (95.3%) had unique genotypes. Taking into account mating type alleles, *AvrLm4-7* alleles and one additional MS marker located 70 kb away from *AvrLm4-7*, the isolates with identical MLGs could be differentiated and shown to be unique genotypes (data not shown).

**Table 4:** Estimates of genetic polymorphism revealed by seven minisatellites and mating type ratios in *AvrLm7* or *avrLm7* populations of *Leptosphaeria maculans* collected from the Grignon field experiment between autumn 2006 and 2008.

Population	*AvrLm7* isolates				*avrLm7* isolates			
	*All*	*2006*	*2007*	*2008*	*All*	*2006*	*2007*	*2008*
N^a^	161	60	46	55	161	52	54	55
Na	9.57	7.71	7.57	6.86	10.14	7.29	7.57	8.86
	±1.89	±1.29	±1.62	±0.88	±1.75	±1.15	±0.95	±1.47
Na Freq. ≥5%	4.14	4.43	4.14	4.43	4.43	4.43	4.14	4.14
	±0.59	±0.61	±0.74	±0.53	±0.48	±0.53	±0.51	±0.67
H	0.66	0.66	0.65	0.66	0.67	0.70	0.65	0.67
	±0.08	±0.08	±0.09	±0.08	±0.07	±0.06	±0.08	±0.07
MLG	156	58	46	54	158	52	54	55
N_MLG_	1.03	1.03	1.00	1.02	1.02	1.00	1.00	1.00
	±0.02	±0.03	±0.00	±0.02	±0.01	±0.00	±0.00	±0.00
*r*_d_	0.00^ns^	−0.01^ns^	0.00^ns^	0.01^ns^	−0.01^ns^	0.00^ns^	−0.02^ns^	−0.02^ns^
Mat1-1/Mat1-2	82/79	30/30	21/25	21/24	89/72	35/17*	33/21	21/34

a N, number of isolates; Na and Na Freq. ≥5%, mean number of different alleles and mean number of different alleles with frequency >0.05, respectively (± standard error); H, unbiased gene diversity averaged across loci (± standard error); MLG, number of multilocus genotypes; N_MLG_, mean number of isolates per genotype (± standard error); *r*_d_, standardized version of the index of association as calculated in Multilocus v3.1; Mat1-1/Mat1-2 is the ratio of the number of isolates carrying mating type 1 (Mat1-1) to isolates carrying mating type 2 (Mat1-2). (*) Significant deviation from a 1∶1 ratio was tested using a χ^2^ with 1 degree of freedom; ns, P>0.05;

* , P<0.05.

Both mating types were present in all isolate samples and occurred in equal frequencies for most of them, except for the sample comprising *avrLm7* isolates collected in autumn 2006, in which a significant deviation (*P* = 0.012) from a 1∶1 ratio was detected (Table 4). This biased frequency may be attributable to a resampling bias or a bias linked to the scarcity of virulent isolates at the beginning of the experiment. The multilocus linkage disequilibrium values (*r_d_*) obtained for all the samples were close to zero and did not deviate significantly from the expectations under the null hypothesis of random mating in all samples (Table 4). These results suggest that there was no genotypic disequilibrium in the samples studied and that recombination regularly generates new *AvrLm7* and *avrLm7* genotypes in the population.

To estimate differentiation between *AvrLm7* and *avrLm7* isolate samples, we firstly tested each pair of samples for heterogeneity in allele frequencies using the Fisher exact test (data not shown). These estimates were consistent with the hypothesis that there was no genetic differentiation between the samples. *F*-statistics also showed no significant population differentiation between them for all loci and overall. Accordingly, the mean *F_ST_* was not significantly different from zero (*P* = 0.51) over loci between *AvrLm7* and *avrLm7* samples. Pair-wise levels of genetic differentiation estimated between all pairs of *AvrLm7* and *avrLm7* samples gave *F_ST_*-estimates which were not significantly different from zero (data not shown). Lastly, hierarchical AMOVA on all samples confirmed the absence of genetic differentiation between *AvrLm7* and *avrLm7* isolates and showed that only 0.04% of the total variance was distributed among populations, with 99.96% within populations (*Φ_ST_* = 0.0004 ; *P* = 0.14).

## Discussion

Rapid adaptation of microbes to control methods (drugs such as antibiotics and fungicides or plant disease resistance) is a very common phenomenon driven by mutation and selection, along with reproduction regime and gene flow that amplify and disperse the new character in the pathogen population [3]. Resistance to drugs can be ascribed to various mechanisms (reduced permeability or enhanced efflux, enzymatic inactivation, alteration or over-expression of the target gene) [26]. In contrast, in simpler gene-for-gene systems representative of numerous plant-pathogen interactions, modification of a target (i.e. the avirulence gene product) allows the pathogen to escape the resistance gene-mediated plant defense responses [27, this study]. Since avirulence gene products are pathogen effectors, how easy it is for the pathogen to modify or delete the target gene depends on the fitness deficit linked with loss or attenuation of its effector function [4], [28], [29]. In addition, the ability of a pathogen to render host resistance ineffective is a function of biological traits that contribute to its “evolutionary potential”, including its reproduction regime, size of populations and dispersal ability [1], [3]. Using a fungal pathogen known to have a high evolutionary potential and a dedicated field experiment, we investigated the “breakdown” of the new resistance gene *Rlm7*, corresponding to the *AvrLm4-7* effector which makes an important contribution to fungal fitness [28], [29]. The establishment of this experiment aimed to address two questions for which little or no information is currently available: (i) what are the initial mutations responsible for rendering ineffective a plant resistance gene at the scale of a single field? (ii) how (and how rapidly) are these mutations generated?

Our data suggest that we have captured all of the possible mutational events existing very early in the process of selection and show that adaptation to selection occurs rapidly through numerous diverse mutational events at the *AvrLm4-7* locus. Almost all mutations lead to gene inactivation or production of a non-functional effector protein. Unexpectedly, in a single 0.25-hectare field we observed all previously reported molecular events (and more) leading to loss of fungal avirulence in world-wide collections of isolates. Most of these mutational events were even observed during the first year of the experiment. These included complete or partial deletion of the gene [10], [14], [27], [30]–[33], amino acid substitutions [14], [27], [34], point deletions and production of truncated proteins [27], and “insertion” of a transposon [33], [35], [36]. In addition, the RIP mutations that commonly occurred, have not been previously reported as an inactivation mechanism for effector genes for pathogens other than *L. maculans* [10], [31]. Three other new phenomena observed were common deletion of an AA dinucleotide, alteration of the gene expression and gene duplication possibly favouring (or responsible for?) RIP mutations.

The speed of “generation” and diversity of mutational events and increased ratio of virulent isolates in the population then raises questions about how these events were generated and dispersed. The first postulate was that strong selection in a large local population would have allowed the emergence of numerous mutational variants. The estimate of *N_e_* obtained here using ABC approaches indeed indicated large effective population size in the field and was comparable to what is described for the few sexually reproducing phytopathogenic fungi for which similar analyses were performed [37], [38]. We then investigated the sequence of mutation events found at the AvrLm4-7 locus following selection and showed that two of the many possible types of mutations were favored over others and varied in frequency over time. RIP was the prevalent mutation pattern at the beginning of the sampling at a time when only few mutants could be found, then followed by large-scale deletions. RIP was initially described in the model ascomycete fungus *Neurospora crassa* as a premeiotic process that efficiently detects and mutates duplicated sequences [39]. In *L. maculans*, the embedding of effector genes in mosaics of RIP-altered TEs and the presence of RIP signatures in the sequence of effector genes indicated that RIP could act on unduplicated sequences to promote gene diversification and that it could “leak” from the neighbouring RIP-affected sequences to generate mutations in single-copy genes [9]. Consistently, the 3′ part of the gene, directly bordered by TEs, is more affected by RIP than its 5′ part and promoter. This hypothesis, however, has to be reconsidered in view of our finding that part of the isolates with RIPped alleles of *AvrLm4-7* probably has two copies of the gene. This might be more consistent with a canonical RIP mechanism, indicating that, at least in part of the cases, gene duplication precedes the action of RIP, thus acting on truly duplicated sequences and may be followed by segregation and deletion of one (or two) copies of the gene. In contrast with this finding, inactivation of the avirulence gene *AvrLm6* by RIP mutations [10], [11] was not associated with duplication of the gene in field isolates, thus substantiating the ‘leaking from neighbouring RIPped regions’ hypothesis [11]. Either leaking from neighbouring RIPped TEs or acting on truly duplicated sequences, RIP is an extraordinary efficient mutation mechanism that affects up to 30% of the G:C pairs of duplicated genes in a single sexual cycle of *N. crassa* [39]. This suggests that RIP mutations can be generated at a very high rate at each sexual cycle of *L. maculans* in the field, i.e. at the beginning of each growing season. In addition, frequency of RIP mutation is increased by the embedding of effector genes in TE-rich blocks of the genome, allowing action of RIP on single-copy genes. Both these data substantiate the importance of genome environment and sexual reproduction to promote an accelerated mutation rate of effector genes (including *AvrLm4-7*) due to RIP [9], [11], which is likely to correspond to the most rapid adaptation to selection. In the second and third years of sampling, large-scale deletions became more common than RIP mutations as an inactivation mechanism illustrating a dynamic process in which many possible virulence alleles are generated, but only a small number eventually survive. While some large-scale deletions may in fact correspond to extremely RIPped alleles as found here for isolate NzT-4, this finding is reminiscent of what we observed when analysing the avrLm1 locus in French populations of the fungus many years after the large-scale use of the *Rlm1* resistance in the field: more than 90% of the virulent isolates had a 260-kb deletion of the gene and its TE-rich environment while only 0.7% of the isolates had an allele with RIP signatures [31]. In *L. maculans*, the presence of four widely expanded TE families representing 25.2% of the *L. maculans* genome [9] provides many targets for mis-pairing between sister chromatids during meiosis and suggests that unequal crossovers lead to production of large deletion/insertion events and production of chromosomes of novel sizes in the progeny [40], [41]. Consistent with clustering of these four families of TEs in AT-isochores containing all currently known avirulence genes of *L. maculans*, large-size deletions were described as the main event leading to virulence at the AvrLm1 and AvrLm6 loci [10], [31], as for the AvrLm4-7 locus (this study).

Analysis of surrounding populations obtained from susceptible *rlm7* cvs. indicated an increase in frequency of virulent *avrLm7* isolates next to the resistant *Rlm7* plot, but not in plots located less than 600 meters away, reflecting the rapid selection of virulent isolates expected in such experimental conditions. The cropping practices used in the field experiment are likely to have two consequences: (i) a large increase in size of the local population due to the lack of rotation and the close contact of the crop with unburied infected residues from the previous years and (ii) an increased rate of sexual reproduction because infected debris were left on the soil surface. RIP mutations, gene duplications and gene deletions, the most common modes of loss of the avirulence at the AvrLm7 locus directly depend upon the ability of the pathogen to undergo sexual mating. Favouring this part of its life cycle along with increase in population size directly impact the ability to generate a large number of virulent progeny and the probability they will be selected for by the resistant cultivars, eventually improving the opportunities for mating between two virulent parent isolates. The (i) wide diversity of RIPped alleles and the lack of increase in the mean number of mutations per allele during the 3 years of the experiment, (ii) the fact that virulent isolates could not be detected in local populations and (iii) the genetic similarities between the virulent and avirulent populations, indicating that virulent and avirulent isolates are part of the same genetic population in which the virulence allele is independently assorting with respect to all of the other genes, all suggest that at least part of the mutations at the AvrLm4-7 locus selected in the experimental field are generated locally within a short-time period and as a result of the large population size and meiotic recombination.

## Materials and Methods

### Field experiments

The field experiment was established at Grignon, France (48° 50′ 28.40″ N latitude; 1° 56′ 13.83″ E longitude) (Figure S1A in Text S1). This location had been used in previous studies to describe *L. maculans* population race structure [22]. The field was a right-angled triangle with a 50 m long base and a 100 m long side. The experiment was started in autumn 2004 and was cropped for four growing seasons (2004–2005 to 2007–2008) as a monoculture of oilseed rape with minimum tillage (chiselling). In normal agronomic practice (e.g. at Grignon before the start of the field experiment), oilseed rape is grown as part of a farm rotation and rarely returns to the same field more than one year in three (Figure S1B in Text S1). Monoculture of oilseed rape without ploughing to bury infected stubble was chosen to increase the amount of annual sexual reproduction and the local population size of *L. maculans*, partly mimicking minimum tillage practices in which infected stubble are left at soil surface. No fungicides were used. The plot was sown with cultivars with *Rlm7* resistance (Roxet in 2004–2005, and Exagone in 2005 to 2008) and was bordered by a 10-meter wide strip cropped with a cultivar without *Rlm7* (Campala) that was used as a trap cultivar [22] (Figure S1B in Text S1). No *Rlm7* cv. had been grown in Grignon fields before the start of the experiment. For comparison purposes, control plots were also assessed for disease severity and occurrence of virulent *avrLm7* isolates of *L. maculans*. These control plots included a series of plots at Grignon cropped with a susceptible cultivar between 2000 and the start of sampling of the experiment (autumn 2006) (Figure S1B in Text S1, Table S1 in Text S1). Other control plots were cropped at Versailles (48° 48′ 27.59″ N latitude; 2° 5′ 12.30″ E longitude), ca. 12 km away from Grignon (Figure S1A in Text S1, Tables S1 and S2 in Text S1) with susceptible cultivars (2000–2007) or with the *Rlm7* cultivar Exagone in autumn 2006 and 2007. These control fields were cropped with the usual agronomical practices of French farmers with ploughing and rotation.

Data collected included severity of stem canker, evaluated at crop maturity using the G2 disease index [42] on 160 randomly chosen plants per cultivar.

### Biological sampling

Due to the life cycle of *L. maculans* in which ascospores are the origin of leaf lesions in autumn, which in turn initiate the systemic colonisation of plants eventually causing the stem canker in the following summer, populations collected from stem canker in the summer of year n, following meiosis, were considered similar to those collected from leaf lesions in the autumn of year n. Isolates were sampled either from leaf lesions (single pycnidial isolates) or from stem cankers (single-ascospore isolates) using methods described by Balesdent et al. [22] and West et al. [43], respectively, during three continuous years corresponding to two cultural cycles (2006–2007; 2007–2008), but to three generations of the fungus (Figure S2 in Text S1).

The number of leaves with lesions collected on the *Rlm7* cultivar ranged between 20 and 200. Typical leaf spot lesions caused by *L. maculans* on the *Rlm7* genotype were rare at the start of the experiment due to the scarcity or absence of virulent isolates (Table S2 in Text S1). Only 24 leaves with phoma leaf spots were found in the whole Grignon plot of Exagone in autumn 2006 and all of them were sampled, with sometimes more than one isolate per leaf sampled (Table 1, Table S2 in Text S1). When more leaf lesions were present (subsequent growing seasons for Exagone, all years for Campala), infected leaves were randomly collected with only one isolate obtained from each individual plant. As the frequency of virulent (*avrLm7*) isolates on Exagone was expected to be small in the first sampling year, 500 distinct leaf lesions were collected from cv. Campala in autumn 2006 (Table S2 in Text S1), so that a frequency ≥0.5% of virulent isolates could be detected with a 95% confidence interval. This number was then decreased to 200 leaves for the second year of the experiment (autumn 2007). Similarly, 100 to 200 infected stems were collected each year from each cultivar. Induction of pseudothecial maturation and isolation of ascospores from infected stems was as described by West et al. [43]. Due to the scarcity of mature pseudothecia, more than one (average three) ejected ascospores were used for isolation from a single stem. Of these, only three isolates or less per stem were analysed using molecular markers (see below).

The frequency of virulent *avrLm7* isolates before the establishment of the field experiment and in the vicinity of the field experiment during the course of the experiment was estimated using a collection of 1233 isolates recovered from leaf lesions or from ascospores on stems of susceptible cultivars including around 100 isolates collected in Grignon in autumn 2006 from two oilseed rape fields located less than 600 m from the field experiment (Table 1, Tables S1 and S2 in Text S1).

### Additional isolates, maintenance of isolates and pathogenicity tests

The reference isolates v23.1.3 (*AvrLm4-AvrLm7*; double avirulent) whose genome sequence is available [9], v23.1.2 (*avrLm4-AvrLm7*; avirulent towards *Rlm7* only), and Nz-T4 (*avrLm4-avrLm7*; double virulent) [44], [45] were used as controls for inoculation tests, and as sequence reference for the AvrLm4-AvrLm7 and avrLm4-AvrLm7 alleles of the *AvrLm4-7* gene [21]. Isolate M3.2, the first *avrLm7* isolate collected in France in 2000 was also included to determine the type of mutation it harbours at the AvrLm4-7 allele [22]. All fungal cultures were maintained on V8-juice agar and conidia were collected from 12–15 day-old cultures according to the procedure described by Ansan-Melayah et al. [46]. *AvrLm4* and *AvrLm7* avirulence/virulence phenotypes were determined following inoculation of 15-days-old *B. napus* cotyledons with 10 µL of 10^7^ mL^−1^ conidia suspension as described by Balesdent et al. [47].

### DNA manipulations and analyses

Genomic DNA was extracted from conidia suspensions using the DNeasy 96 Plant Kit and the QIAGEN BioRobot 3000 in accordance with the manufacturer's recommendations. PCR primers used for mating type amplification, *AvrLm4-7* analyses and minisatellite analyses were designed with PRIMER 3 [48] and are described in Supplementary Table S4. *AvrLm4-7* was amplified using (i) “external” primers spanning part of the promoter region and part of the 3′ UTR and generating a 1434 bp fragment and (ii) “internal” primers located within the coding sequence, between the ATG and intron and spanning 478 bp (Figure 3A). Standard PCR were performed in an Eppendorf Mastercycler EP Gradient thermocycler (Eppendorf, Le Pecq, France), with 30 cycles of 94°C for 30 s, 30 s of hybridization with variable hybridization temperatures, 72°C for 80 s, with a final extension at 72°C of variable duration (Table S4 in Text S1). Sequencing was performed on PCR products using a Beckman Coulter CEQ 8000 automated sequencer (Beckman Coulter, Fullerton, CA, USA) according to the manufacturer's instructions. Primers for sequencing (Figure 3A) were chosen so that all bases of the gene, including the 5′ UTR and 128 bp of the promoter region were independently read two or three times. Sequences were compared following sequence alignment using MULTALIN and CLUSTALX [49], [50]. Automated analysis of RIP in *AvrLm4-7* alleles was done using RIPCAL (http://www.sourceforge.net/projects/ripcal), a software tool that computes RIP indexes and performs alignment-based analyses [25].

For high-throughput identification of *AvrLm4-7* allelic variants before allele sequencing (when relevant), High Resolution Melting PCR (HRM PCR) was used as an alternative to sequencing, using qPCR 7500 Fast Real-Time PCR equipment (Applied Biosystems). A set of control isolates with known *AvrLm4-7* sequences was included in each HRM-PCR run. Melting curves were analysed with High Resolution Melting software v2.0 (Applied Biosystems).

To recover DNA sequences flanking *AvrLm4-7*, Thermal Asymmetric Interlaced PCR (TAIL-PCR) was used, following the design of nested *AvrLm4-7* sequence-specific primers (Table S4 in Text S1). Arbitrary Degenerated (AD) primers used in association with *AvrLm4-7* primers were AD1, AD2 and AD3 [51] (Table S4 in Text S1), with AD2 used for the two subsequent rounds of PCR amplification. First and second rounds of TAIL PCR were done as described by Liu & Whittier [51]. Secondary TAIL-PCR products were purified using the Nucleospin Extract II purification Kit (Macherey-Nagel, Hoerd, Fr) and were used either for the third round of TAIL PCR, whenever the amount of amplified product was insufficient, or as template for DNA sequencing using the specific tertiary border primer Tail-GD3 as a sequencing primer (Figure 3A). To validate the TAIL-PCR results, primers were designed with PRIMER 3 from the TAIL-PCR sequence product and used to PCR-amplify the corresponding sequence from genomic DNA of the corresponding isolate, with v23.1.3 as a negative control.

For Southern blots, mycelia from the isolates grown in liquid Fries medium for two weeks were harvested by filtration, freeze-dried, ground to a fine powder, and DNA extraction was done as described by Balesdent et al. [52]. Southern blot analysis was performed on *Xba*I or *Spe*I or *Hpa*I restricted genomic DNA (10 µg), size-fractionated on 0.8% w/v agarose gels and transferred to positively charged nylon membrane (Qbiogen) according to standard protocols. A 459-bp probe was generated by PCR using the AvrLm4-7Int-F and AvrLm4-7ext-R primers (Table S4 in Text S1) and gel purified after electrophoresis using the NucleoSpin Plasmid QuickPure kit (Machery-Nagel). Preparation of a [α-^32^P]dCTP probe was performed using the random priming Ready-To-Go DNA labelling beads kit (GE Healthcare). High stringency hybridization (65°C) was done using standard protocols.

For Quantitative RT-PCR, cotyledons of cv. Westar (susceptible control) were inoculated and sampled 7 days after inoculation. Total RNA extraction and single-strand cDNA synthesis were performed as described by Fudal et al. [19]. Inoculation and RNA extraction were repeated twice. Water and RNA from plants inoculated with isolate G07-E441, lacking the *AvrLm4-7* gene, were used as negative controls. Primers for *AvrLm4-7* amplification were as described by Parlange et al. [21]. qRT-PCR was performed using 7700 real-time PCR equipment (Applied Biosystems, Foster City, CA, USA) and ABsolute SYBR Green ROX dUTP Mix (ABgene, Courtaboeuf, France), as described by Fudal et al. [19]. Actin was used as a constitutive reference gene.

### Markers for population genetics analyses

A multiplex PCR was used to characterize the distribution of the Mat1-1 and Mat1-2 alleles in the collection of isolates [53]. Seven genetically independent minisatellite markers (Table S4 in Text S1) were used for population genetic analyses [54]. For each isolate, the allele sizes were determined using quantity one 1-D Analysis software (BioRad, Marnes-la-Coquette, France) by comparison with band sizes of the 1-kb^+^ ladder (Invitrogen, Cergy Pontoise, France) and internal control with known allele size and known number of repeats of the core motif (i.e., the sequenced reference v23.1.3 isolate). Data were scored as the number of repeat units for each isolate and each minisatellite locus.

### DATA analyses: population genetics

ABC methods implemented in the DIYABC program v1.0.4.39 [55] were used to estimate the effective population size (*Ne*) of the *L. maculans* populations sampled in 2006, 2007 and 2008 from the susceptible cv. Campala. For the genetic parameters, the Generalized Stepwise Mutation (GSM) model was used to simulate mutations at the minisatellite loci and the prior interval specifications for the mean mutation rate were as described in Dilmaghani et al. [54]. A total of 1,000,000 data sets were simulated to generate a reference table. This reference table comprised summary statistics (e.g. genetic diversity per sample and genetic distance between samples) that enable estimation of the posterior distributions of the demographic parameters, under a given scenario, using comparisons between simulated and observed data sets. We used DIYABC to estimate effective population size under a simple scenario corresponding to one population, from which several samples had been taken over three consecutive generations (three events of sexual reproduction). The summary statistics used were mean number of alleles per locus, mean genetic diversity [56], mean variance in allele size, genetic differentiation between pairwise groups (*F_ST_*, [57]) and genetic distances (σμ)^2^ [58]. A local linear regression on the 1% simulated data sets closest to the observed data sets was then used to estimate the posterior distribution of the parameters. A generation time of 1 year was assumed, based on biological and epidemiological studies [24], [59].

The frequency of each molecular event leading to virulence (*avrLm7*) was calculated in the different isolate samples. Where more than one isolate was recovered from a single plant, as with isolates obtained from stem residues, a sub-sample was obtained by random selection of a single isolate from each individual plant. The molecular event for each isolate sampled was then recorded and the frequency of each molecular event was calculated on the basis of this sub-sampling. This randomized sub-sampling was repeated 10,000 times, and the mean frequency for each molecular event calculated. The resulting frequencies of each type of event were compared over the three years using the Approximate Pearson's Chi squared test with 20,000 randomizations as implemented in XLSTAT v2010.5.01.

To analyse minisatellite variability, the software FSTAT version 2.9 [60] was used to compute allele frequencies, number of alleles per minisatellite (A), number of private alleles and Nei's gene diversity (H) [61] at each locus and over all loci, within and over the samples. Tests for differences between groups of samples comprising *AvrLm7* or *avrLm7* isolates for polymorphism statistics were based on two-sided permutation tests (15,000 permutations) and performed using FSTAT.

Linkage disequilibrium was evaluated using two different approaches. First, minisatellites were tested pairwise within and across samples using the genotypic disequilibrium test in Genepop [62]. The statistical significance of each pairwise test of linkage disequilibrium was tested by Fisher's exact test. The associations of alleles among different loci were also estimated with the standardized version of the index of association *r_d_*, using MULTILOCUS [63]. The significance of *r_d_* was established by comparing the observed value to the distribution obtained from 1000 randomizations with alleles at each locus being resampled without replacement to simulate the effect of random mating. The hypothesis of random mating was tested as follows: the distribution of mating types was compared to the 1∶1 ratio expected under random mating for a haploid fungus using χ^2^ tests. Genetic structure was analysed with standard FST coefficients of population differentiation, which were calculated and tested for significance using 1000 permutations using FSTAT. To further analyse population differentiation, heterogeneity of allele frequencies among samples was tested for each locus using the Fisher exact test in the GENEPOP program [63]. Genetic differentiation amongst samples was examined using an analysis of molecular variance (AMOVA) in Arlequin v3.5 [64].

## Supporting Information

Text S1 Additional information regarding experimental field location, sampling design, sampling collections, Southern blots, q-RT-PCR analyses and PCR primers.
(DOC)

## References

[1] Stukenbrock EH, McDonald BA (2008) The origins of plant pathogens in agro-ecosystems. Annu Rev Phytopathol 46: 75–100. 18680424 10.1146/annurev.phyto.010708.154114

[2] Hammond-Kosack KE, Kanyuka K (2007) Resistance genes (*R* genes) in plants. In: Encyclopedia of Life Sciences (ELS). Chichester: John Wiley & Sons, Ltd. DOI: 10.1002/9780470015902.a0020119.

[3] McDonald BA, Linde C (2002) The population genetics of plant pathogens and breeding for durable resistance. Euphytica 12: 163–180.

[4] Leach JE, Vera Cruz CM, Bai J, Leung H (2001) Pathogen fitness penalty as a predictor of durability of disease resistance genes. Ann Rev Phytopathol 39: 187–224. 11701864 10.1146/annurev.phyto.39.1.187

[5] Aubertot JN, West JS, Bousset-Vaslin L, Salam MU, Barbetti MJ, et al (2006) Improved management for durable disease control: a case study of phoma stem canker of oilseed rape (*Brassica napus*). Eur J Plant Pathol 114: 91–106.

[6] Flor HH (1956) The complementary genic systems in flax and flax rust. Adv Genet 8: 275–296.

[7] Jones JD, Dangl JL (2006) The plant immune system. Nature 444: 323–329. 17108957 10.1038/nature05286

[8] Schmidt SM, Panstruga R (2011) Pathogenomics of fungal plant parasites: what have we learnt about pathogenesis? Curr Op Plant Biol 14: 392–399. 10.1016/j.pbi.2011.03.006 21458359

[9] Rouxel T, Grandaubert J, Hane JK, Hoede C, van de Wouw AP, et al (2011) Effector diversification within compartments of the *Leptosphaeria maculans* genome affected by Repeat Induced Point mutations. Nat Commun 2: 202. 21326234 10.1038/ncomms1189 PMC3105345

[10] Fudal I, Ross S, Brun H, Besnard AL, Ermel M, et al (2009) Repeat-Induced Point mutation (RIP) as an alternative mechanism of evolution toward virulence in *Leptosphaeria maculans* . Mol Plant-Microbe Interact 22: 932–941. 19589069 10.1094/MPMI-22-8-0932

[11] van de Wouw A, Cozijnsen AJ, Hane JK, Brunner PC, McDonald BA, et al (2010) Evolution of linked avirulence effectors in *Leptosphaeria maculans* is affected by genomic environment and exposure to resistance genes in host plants. PLoS Pathogens 6: e1001180. 21079787 10.1371/journal.ppat.1001180 PMC2973834

[12] Rouxel T, Balesdent MH (2005) The stem canker (blackleg) fungus, *Leptosphaeria maculans*, enters the genomic era. Mol Plant Pathol 6: 225–241. 20565653 10.1111/j.1364-3703.2005.00282.x

[13] Stergiopoulos I, de Wit PJGM (2009) Fungal effector proteins. Annu Rev Phytopathol 47: 233–263. 19400631 10.1146/annurev.phyto.112408.132637

[14] Schürch S, Linde CC, Knogge W, Jackson LF, McDonald BA (2004) Molecular population genetic analysis differentiates two virulence mechanisms of the fungal avirulence gene *NIP1* . Mol Plant-Microbe Interact 17: 1114–1125. 15497404 10.1094/MPMI.2004.17.10.1114

[15] Ansan-Melayah D, Rouxel T, Bertrandy J, Letarnec B, Mendes-Pereira E, et al (1997) Field efficiency of *Brassica napus* specific resistance correlates with *Leptosphaeria maculans* population structure. Eur J Plant Pathol 103: 835–841.

[16] Delourme R, Chèvre AM, Brun H, Rouxel T, Balesdent MH, et al (2006) Major gene and polygenic resistance to *Leptosphaeria maculans* in oilseed rape (*Brassica napus*). Eur J Plant Pathol 114: 41–52.

[17] Li H, Sivasithamparam K, Barbetti MJ (2003) Breakdown of a *Brassica rapa* ssp. *sylvestris* single dominant blackleg resistance gene in *B. napus* rapeseed by *Leptosphaeria maculans* field isolates in Australia. Plant Dis 87: 752. 10.1094/PDIS.2003.87.6.752A 30812879

[18] Rouxel T, Penaud A, Pinochet X, Brun H, Gout L, et al (2003) A ten-year survey of populations of *Leptosphaeria maculans* in France indicates a rapid adaptation towards the *Rlm1* resistance gene in oilseed rape. Eur J Plant Pathol 109: 871–881.

[19] Fudal I, Ross S, Gout L, Blaise F, Kuhn ML, et al (2007) Heterochromatin-like regions as ecological niches for avirulence genes in *Leptosphaeria maculans* genome: map-based cloning of *AvrLm6* . Mol Plant-Microbe Interact 20: 459–470. 17427816 10.1094/MPMI-20-4-0459

[20] Gout L, Fudal I, Kuhn ML, Blaise F, Cattolico L, et al (2006) Lost in the middle of nowhere: the *AvrLm1* avirulence gene of *Leptosphaeria maculans* . Mol Microbiol 60: 67–80. 16556221 10.1111/j.1365-2958.2006.05076.x

[21] Parlange F, Daverdin G, Fudal I, Kuhn ML, Balesdent MH, et al (2009) *Leptosphaeria maculans* avirulence gene *AvrLm4-7* confers a dual recognition specificity by the *Rlm4* and *Rlm7* resistance genes of oilseed rape, and circumvents *Rlm4*-mediated recognition through a single amino acid change. Mol Microbiol 71: 851–863. 19170874 10.1111/j.1365-2958.2008.06547.x

[22] Balesdent MH, Louvard K, Pinochet X, Rouxel T (2006) A large scale survey of races of *Leptosphaeria maculans* occurring on oilseed rape in France. Eur J Plant Pathol 114: 53–65.

[23] Marcroft SJ, Sprague SJ, Pymer SJ, Salisbury PA, Howlett BJ (2004) Crop isolation, not extended rotation length, reduces blackleg (*Leptosphaeria maculans*) severity of canola (*Brassica napus*) in south-eastern Australia. Aust J Exp Agric 44: 601–606.

[24] West JS, Kharbanda PD, Barbetti MJ, Fitt BDL (2001) Epidemiology and management of *Leptosphaeria maculans* (phoma stem canker) on oilseed rape in Australia, Canada and Europe. Plant Pathol 50: 10–27.

[25] Hane JK, Oliver RP (2008) RIPCAL: a tool for alignment-based analysis of repeat-induced point mutations in fungal genomic sequences. BMC Bioinformatics 9: 478. 19014496 10.1186/1471-2105-9-478 PMC2621366

[26] Ma Z, Michailides T (2005) Advances in understanding molecular mechanisms of fungicide resistance and molecular detection of resistant genotypes in phytopathogenic fungi. Crop Protect 24: 853–863.

[27] Stergiopoulos I, De Kock MJ, Lindhout P, de Wit PJ (2007) Allelic variation in the effector genes of the tomato pathogen *Cladosporium fulvum* reveals different modes of adaptive evolution. Mol Plant-Microbe Interact 20: 1271–1283. 17918629 10.1094/MPMI-20-10-1271

[28] Huang YJ, Li ZQ, Evans N, Rouxel T, Fitt BDL, et al (2006) Fitness cost associated with loss of the *AvrLm4* avirulence function in *Leptosphaeria maculans* (phoma stem canker of oilseed rape). Eur J Plant Pathol 114: 77–89.

[29] Huang YJ, Balesdent MH, Li ZQ, Evans N, Rouxel T, et al (2010) Fitness cost of virulence differs between the *AvrLm1* and *AvrLm4* loci in *Leptosphaeria maculans* (phoma stem canker of oilseed rape). Eur J Plant Pathol 126: 279–291.

[30] Farman MK (2007) Telomeres in the rice blast fungus *Magnaporthe oryzae*: the world of the end as we know it. FEMS Microbiol Lett 273: 125–132. 17610516 10.1111/j.1574-6968.2007.00812.x

[31] Gout L, Kuhn ML, Vincenot L, Bernard-Samain S, Cattolico L, et al (2007) Genome structure impacts molecular evolution at the *AvrLm1* avirulence locus of the plant pathogen *Leptosphaeria maculans* . Environ Microbiol 9: 2978–2992. 17991027 10.1111/j.1462-2920.2007.01408.x

[32] Rep M, van der Does HC, Meijer M, van Wijk R, Houterman PM, et al (2004) A small, cysteine-rich protein secreted by *Fusarium oxysporum* during colonization of xylem vessels is required for *I-3*-mediated resistance in tomato. Mol Microbiol 53: 1373–83. 15387816 10.1111/j.1365-2958.2004.04177.x

[33] Zhou E, Jia Y, Singh P, Correll JC, Lee FN (2007) Instability of the *Magnaporthe oryzae* avirulence gene *AVR-Pita* alters virulence. Fungal Genet Biol 44: 1024–1034. 17387027 10.1016/j.fgb.2007.02.003

[34] Ellis JG, Lawrence GJ, Dodds PN (2007) Further analysis of gene-for-gene disease resistance specificity in flax. Mol Plant Pathol 8: 103–109. 20507482 10.1111/j.1364-3703.2006.00375.x

[35] Fudal I, Bohnert HU, Tharreau D, Lebrun MH (2005) Transposition of MINE, a composite retrotransposon, in the avirulence gene *ACE1* of the rice blast fungus *Magnaporthe grisea* . Fungal Genet Biol 42: 761–772. 15978851 10.1016/j.fgb.2005.05.001

[36] Kang S, Lebrun MH, Farrall L, Valent B (2001) Gain of virulence caused by insertion of a *Pot3* transposon in a *Magnaporthe grisea* avirulence gene. Mol Plant-Microbe Interact 14: 671–674. 11332731 10.1094/MPMI.2001.14.5.671

[37] Barrès B, Carlier J, Seguin M, Fenouillet C, Cilas C, et al (2012) Understanding the recent colonization history of a plant pathogenic fungus using population genetic tools and Approximate Bayesian Computation. Heredity E-pub ahead of print. doi:10.1038/hdy.2012.37. 10.1038/hdy.2012.37 PMC3498996 22828899

[38] Zhan J, McDonald BA (2004) The interaction among evolutionary forces in the pathogenic fungus *Mycosphaerella graminicola* . Fungal Genet Biol 41: 590–599. 15121082 10.1016/j.fgb.2004.01.006

[39] Galagan JE, Selker EU (2004) RIP: the evolutionary cost of genome defense. Trends Genet 9: 417–423. 10.1016/j.tig.2004.07.007 15313550

[40] Leclair S, Ansan-Melayah D, Rouxel T, Balesdent MH (1996) Meiotic behaviour of the minichromosome in the phytopathogenic ascomycete *Leptosphaeria maculans* . Curr Genet 30: 541–548. 8939816 10.1007/s002940050167

[41] Plummer KM, Howlett BJ (1995) Inheritance of chromosomal length polymorphisms in the ascomycete *Leptosphaeria maculans* . Mol Gen Genet 247: 416–422. 7770048 10.1007/BF00293142

[42] Aubertot JN, Schott JJ, Penaud A, Brun H, Doré T (2004) Methods for sampling and assessment in relation to the spatial pattern of phoma stem canker (*Leptosphaeria maculans*) in oilseed rape. Eur J Plant Pathol 110: 183–192.

[43] West JS, Balesdent MH, Rouxel T, Narcy JP, Huang YJ, et al (2002) Colonization of winter oilseed rape tissues by A/Tox^+^ and B/Tox^0^ *Leptosphaeria maculans* (phoma stem canker) in France and England. Plant Pathol 51: 311–321.

[44] Balesdent MH, Attard A, Ansan-Melayah D, Delourme R, Renard M, et al (2001) Genetic control and host range of avirulence toward *Brassica napus* cultivars Quinta and Jet Neuf in *Leptosphaeria maculans* . Phytopathology 91: 70–76. 18944280 10.1094/PHYTO.2001.91.1.70

[45] Balesdent MH, Attard A, Kuhn ML, Rouxel T (2002) New avirulence genes in the phytopathogenic fungus *Leptosphaeria maculans* . Phytopathology 92: 1122–1133. 18944223 10.1094/PHYTO.2002.92.10.1122

[46] Ansan-Melayah D, Balesdent MH, Buée M, Rouxel T (1995) Genetic characterization of *AvrLm1*, the first avirulence gene of *Leptosphaeria maculans* . Phytopathology 85: 1525–1529.

[47] Balesdent MH, Barbetti MJ, Li H, Sivasithamparam K, Gout L, et al (2005) Analysis of *Leptosphaeria maculans* race structure in a worldwide collection of isolates. Phytopathology 95: 1061–1071. 18943304 10.1094/PHYTO-95-1061

[48] Rozen S, Skaletsky H (2000) Primer3 on the WWW for general users and for biologist programmers. In: Krawetz S, Misener S (eds) Bioinformatics Methods and Protocols: Methods in Molecular Biology. Totowa, NJ: Humana Press. pp 365–386. 10.1385/1-59259-192-2:365 10547847

[49] Corpet F (1988) Multiple sequence alignment with hierarchical-clustering. Nucleic Acids Res 16: 10881–10890. 2849754 10.1093/nar/16.22.10881 PMC338945

[50] Thompson JD, Gibson TJ, Plewniak F, Jeanmougin F, Higgins DG (1997) The CLUSTAL_X windows interface: flexible strategies for multiple sequence alignment aided by quality analysis tools. Nucleic Acids Res 25: 4876–4882. 9396791 10.1093/nar/25.24.4876 PMC147148

[51] Liu YG, Whittier RF (1995) Thermal asymmetric interlaced PCR-automatable amplification and sequencing of insert end fragments from p1 and YAC clones for chromosome walking. Genomics 25: 674–681. 7759102 10.1016/0888-7543(95)80010-j

[52] Balesdent MH, Jedryczka M, Jain L, Mendes-Pereira E, Bertrandy J, et al (1998) Conidia as a substrate for Internal Transcribed Spacer-based PCR identification of members of the *Leptosphaeria maculans* species complex. Phytopathology 88: 1210–1217. 18944856 10.1094/PHYTO.1998.88.11.1210

[53] Cozijnsen AJ, Howlett BJ (2003) Characterisation of the mating type locus of the plant pathogenic ascomycete *Leptosphaeria maculans* . Curr Genet 43: 351–357. 12679880 10.1007/s00294-003-0391-6

[54] Dilmaghani A, Gladieux P, Gout L, Giraud T, Brunner PC, et al (2012) Migration patterns and changes in population biology associated with the worldwide spread of the oilseed rape pathogen *Leptosphaeria maculans* . Mol Ecol 21: 2519–33. 22439871 10.1111/j.1365-294X.2012.05535.x

[55] Cornuet JM, Ravigné V, Estoup A (2010) Inference on population history and model checking using DNA sequence and microsatellite data with the software DIYABC (v1.0). BMC Bioinformatics 11: 401. 20667077 10.1186/1471-2105-11-401 PMC2919520

[56] Nei M (1978) Estimation of average heterozygosity and genetic distance from a small number of individuals. Genetics 89: 583–590. 17248844 10.1093/genetics/89.3.583 PMC1213855

[57] Weir BS, Cockerham CC (1984) Estimating F-statistics for the analysis of population structure. Evolution 38: 1358–1370. 28563791 10.1111/j.1558-5646.1984.tb05657.x

[58] Goldstein DB, Ruiz Linares A, Cavalli-Sforza LL, Feldman MW (1995) An evaluation of genetic distances for use with microsatellite loci. Genetics 139: 463–471. 7705647 10.1093/genetics/139.1.463 PMC1206344

[59] Rouxel T, Balesdent MH (2010) Avirulence Genes. In: Encyclopedia of Life Sciences (ELS). Chichester: John Wiley & Sons, Ltd. DOI: 10.1002/9780470015902.a0021267.

[60] Goudet J (2001) FSTAT, a program to estimate and test gene diversities and fixation indices (version 2.9.3), updated from Goudet J (1995) FSTAT (version 1.2): a computer program to calculate F-statistics. J Hered 86: 485–486.

[61] Nei M (1973) Analysis of gene diversity in subdivided populations. Proc Natl Acad Sci USA 12: 3321–3323. 10.1073/pnas.70.12.3321 PMC427228 4519626

[62] Raymond M, Rousset F (1995) GENEPOP (version-1.2) - Population-genetics software for exact tests and ecumenicism. J Heredity 86: 248–249.

[63] Agapow PM, Burt A (2001) Indices of multilocus linkage disquilibrium. Mol Ecol Notes 1: 101–102.

[64] Excoffier L, Laval G, Schneider S (2005) Arlequin ver. 3.0: an integrated software package for population genetics data analysis. Evol Bioinf Online 1: 47–50. PMC2658868 19325852

