# Supplementary material for: Genome Structure and Reproductive Behaviour Influence the Evolutionary Potential of a Fungal Phytopathogen
Source: PLoS Pathog. 2012 Nov 8;8(11):e1003020. doi: 10.1371/journal.ppat.1003020 (PMC3493481; doi:10.1371/journal.ppat.1003020)
Supplement: Text S1 — Additional information regarding experimental field location, sampling design, sampling collections, Southern blots, q-RT-PCR analyses and PCR primers. (DOC) [file ppat.1003020.s001.doc]

**Genome Structure and Reproductive Behaviour Influence the Evolutionary Potential of a Fungal Phytopathogen**

**Guillaume Daverdin, Thierry Rouxel, Lilian Gout, Jean-Noël Aubertot, Isabelle Fudal, Michel Meyer, Francis Parlange, Julien Carpezat & Marie-Hélène Balesdent^*^**

**Supplementary Figures**

**Figure S1. Location of oilseed rape (OSR) experimental sites and fields. A.** Map of the location of the two experimental sites, Grignon and Versailles. **B.** In Grignon, location of the experimental fields grown with oilseed rape in 2000-2006. Red plot, the experimental field cultivated with *Rlm7* as a single crop from 2004 to 2008. Black plot, location of the rows grown with the trap cultivar Campala (devoid of *Rlm7*) from 2006 to 2008. Yellow, light green, pink, dark blue, orange, light blue, white, and dark green plots, fields grown with OSR in 2000, 2001, 2002, 2003, 2004, 2005, 2006 and 2007, respectively. The red plot was the only one cultivated with *Rlm7* during the whole 2000-2008 period. The stars indicate the location of the fields where additional samplings were performed on non-*Rlm7* varieties in autumn 2006. Fields that were not cropped with oilseed rape were grown with cereals (maize, wheat or barley).


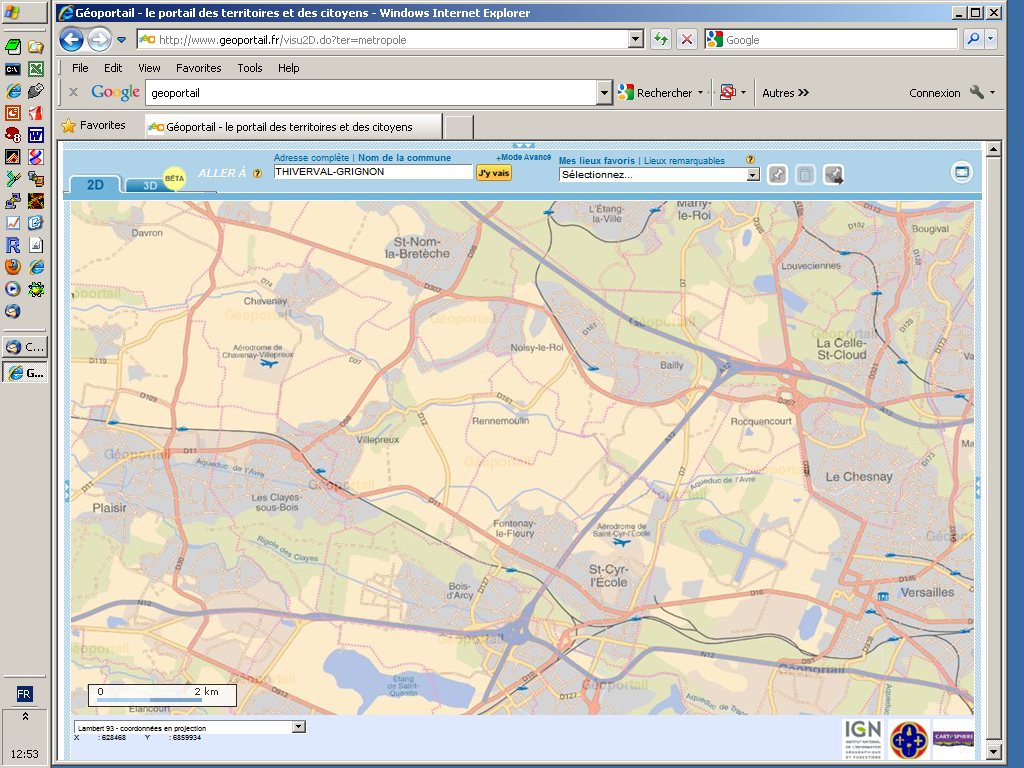


Grignon site

Versailles site


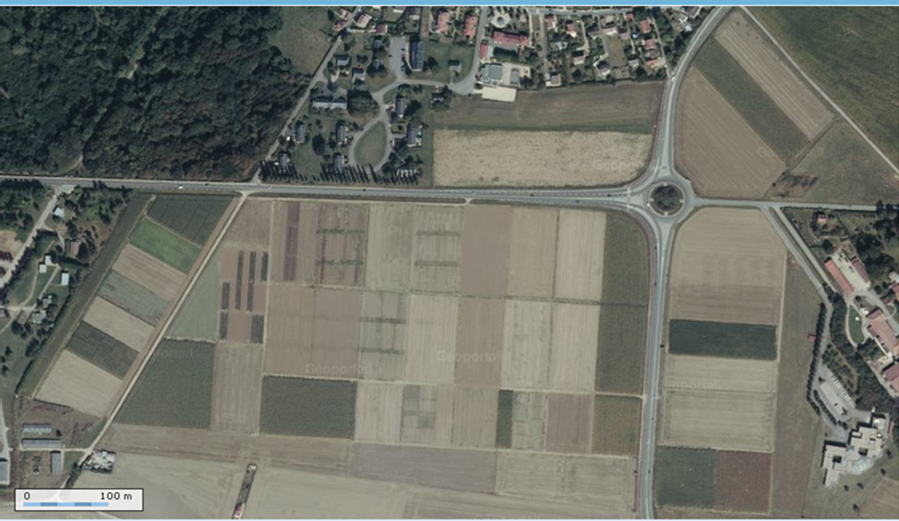

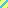

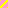

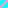

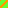

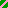

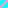

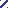

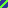


*****


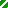


*****

A

B

**Figure S2**. **Schematic representation of the sampling scheme used to obtain virulent *avrLm7* isolates of *Leptosphaeria maculans* from *Rlm7* plant genotypes and control surrounding populations from a susceptible cultivar.** For a given growing season, samplings were done from leaf symptoms in autumn (single-conidium isolates) and from stems in summer (single-ascospore isolates), following the systemic colonisation of plants by the fungus and setting up of its sexual stage on stem residues. Due to the life cycle of *L. maculans* in which ascospores are the origin of leaf lesions in autumn, which in turn initiate the systemic colonisation of plants eventually causing the stem canker in summer, populations collected from stem canker at year n, following meiosis, are the same generation than those collected from leaf lesions at autumn of year n. The two seasons of sampling (2006-2007 and 2007-2008) thus correspond to three generations of the fungus.


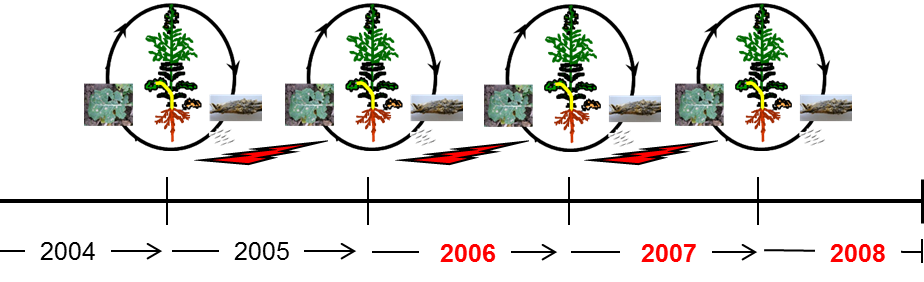


2^nd^ generation

(“2007”)

1^st^ generation

(“2006”)

3^rd^ generation

(“2008”)

Year

Growing

season

**2007-2008**

**2004-2005**

**2005-2006**

**2006-2007**

**Figure S3. Q-RT-PCR analysis of the expression of the *AvrLm4-7* gene of virulent, *avrLm7* isolates *in planta* seven days post inoculation.** Virulent isolates (in black) showed either an unaltered coding sequence (G07-C286, G08-E1023 and G08-E1025), complete gene deletion (G07-E441), or single non synonymous mutations (R100P; G07-E1055 and S112R; G06-E202). Avirulent isolates (grey) have either the *AvrLm4-AvrLm7* (v23.1.3) or the *avrLm4-AvrLm7* (v23.1.2) allele. Each data point is the average of three technical and two biological repeats, expect for G07-E1055, G06-E202 and v23.1.2 with only one biological repeat. *AvrLm4-7* expression was analysed by qRT-PCR and measured relatively to actin. Standard error of mean normalized expression relative to v23.1.3 is indicated by vertical bars.

**Supplementary tables**

**Supplementary Table S1. Number and origin of isolates obtained and phenotyped for virulence towards *Rlm4* and *Rlm7*: collections obtained from cvs. devoid of *Rlm7* before the setting up of the Grignon experimental field or close to it in the course of the experiment.**

| Year | Location | Cultivar sampled^a^ | Organ sampled | No of leaves or stems collected^b^ | No of isolates obtained | No of *L. maculans* isolates phenotyped^c^ | Interaction phenotype (%)^d^ | | |
| --- | --- | --- | --- | --- | --- | --- | --- | --- | --- |
|  |  |  |  |  |  |  | *avrLm4-avrLm7* | *avrLm4-AvrLm7* | *AvrLm4-AvrLm7* |
| 2000 | Grignon | Drakkar | Leaves | 100 | 100 | 100 | 0.00 | 89.00 | 11.00 |
| 2000 | Versailles ^e^ | Drakkar | Leaves | 100 | 87 | 87 | 0.00 | 80.46 | 19.54 |
| 2002 | Grignon | Bristol | Stem | 47 | 98 | 98 | 0.00 | 82.65 | 17.35 |
| 2002 | Grignon | Pollen | Stem | 36 | 93 | 93 | 0.00 | 100.00 | 0.00 |
| 2002 | Versailles | Darmor | Leaves | 46 | 46 | 46 | 0.00 | 93.48 | 6.52 |
| 2002 | Versailles | Mohican | Leaves | 49 | 49 | 49 | 0.00 | 97.96 | 2.04 |
| 2003 | Versailles | Mohican | Stem | 29 | 29 | 29 | 3.45 | 86.21 | 10.34 |
| 2003 | Versailles | Mohican | Leaves | 17 | 17 | 17 | 0.00 | 94.12 | 5.88 |
| 2004 | Versailles | Mohican | Stem | 210 | 210 | 210 | 0.00 | 87.62 | 12.38 |
| 2006 | Grignon, field 2 ^f^ | Grizzly | Leaves | 100 | 100 | 47 | 0.00 | 100.00 | 0.00 |
| 2006 | Grignon, field 3^e^ | Campala | Leaves | 100 | 84 | 46 | 0.00 | 93.48 | 6.52 |

^a^ Drakkar is an oilseed rape cv. devoid of any *Rlm* gene; Mohican, Campala and Darmor contain *Rlm9*, Bristol *Rlm2* and *Rlm9*, Pollen *Rlm4*, and Grizzly *Rlm2* and *Rlm3.*

^b^ One single-conidium isolate was obtained from each leaf lesion, whereas more than one single-ascospore isolate could be obtained from each stem residues.

^c^ Excluding isolates belonging to the other species of the species complex, *Leptosphaeria biglobosa*, and those for which the interaction phenotype could not be clearly established.

^d^ Values are percentages in each population of isolates with the following phenotypes:*avrLm4-avrLm7*, isolates virulent on both *Rlm4* and *Rlm7*; *avrLm4-AvrLm7*, isolates avirulent on *Rlm7* only; *AvrLm4-AvrLm7*, isolates avirulent on both *Rlm4* and *Rlm7.*

^e^ Control fields were cropped with susceptible cvs. at Versailles, ca. 12 km away from Grignon. These fields were cropped with the usual agronomical practices of French farmers with ploughing and rotation.

^f^ Collected from fields located less than 600 m away from the experimental trial.

**Supplementary Table S2. Number and origin of isolates collected in the experimental field and phenotyped for virulence towards *Rlm4* and *Rlm7.***

| Year | Location | Cultivar sampled^a^ | Organ Sampled | No of leaves or stems collected^b^ | No of isolates obtained | No of *L. maculans* isolates phenotyped^c^ | No (%) of isolates in each phenotypic class^d^ | | | |
| --- | --- | --- | --- | --- | --- | --- | --- | --- | --- | --- |
|  |  |  |  |  |  |  | *avrLm4-avrLm7* | *avrLm4-AvrLm7* | *AvrLm4-AvrLm7* | nd |
| 2006 | Grignon | Campala | Leaves | 500 | 500 | 445 | 41 (9.3%) | 384 (86.3%) | 12 (2.7%) | 8 (1.8%) |
|  | Grignon | Exagone | Leaves | 24 | 35 | 35 | 14 (40%) | 21 (60%) | 0 (0%) | 0 (0%) |
|  | Versailles | Campala | Leaves | 500 | 500 | 246 | 0 (0%) | 242 (98.4%) | 4 (1.7%) | 0 (0%) |
| 2007 | Grignon | Campala | Stem | 100 | 43 | 40 | 9 (22.5%) | 31 (77.5%) | 0 (0%) | 0 (0%) |
|  | Grignon | Exagone | Stem | 100 | 108 | 101 | 85 (84.2%) | 16 (15.9%) | 0 (0%) | 0 (0%) |
|  | Versailles | Campala | Stem | 100 | 7 | 5 | 1 (20%) | 4 (80%) | 0 (0%) | 0 (0%) |
|  | Grignon | Campala | Leaves | 200 | 184 | 178 | 56 (31.5%) | 118 (66.3%) | 3 (1.7%) | 1 (0.6%) |
|  | Grignon | Exagone | Leaves | 200 | 191 | 187 | 176 (94.2%) | 10 (5.4%) | 1 (0.6%) | 0 (0%) |
|  | Versailles | Campala | Leaves | 200 | 200 | 160 | 1 (0.7%) | 153 (95.7%) | 3 (1.9%) | 3 (1.9%) |
| 2008 | Grignon | Campala | Stem | 200 | 260 | 235 | 85 (36.2%) | 139 (59.2%) | 10 (4.3%) | 1 (0.5%) |
|  | Grignon | Exagone | Stem | 200 | 523 | 355 | 336 (94.7%) | 12 (3.4%) | 7 (2.0%) | 0 (0%) |
| Total |  |  | |  | 2551 | 1987 | 804 | 1130 | 40 | 13 |

^a^ Exagone is an oilseed rape cv. harbouring the resistance gene *Rlm7*, whereas Campala is devoid of it.

^b^ Each single-conidium isolate was obtained from a single leaf lesion originating from distinct plants, except for Exagone/leaves 2006 for which some isolates may come from different leaf lesions on the same leaf/plant, because very few leaf lesions were found. In contrast, more than one single-ascospore isolate could be obtained from each stem residues.

^c^ Excluding isolates belonging to the *L. biglobosa* species, and those for which the interaction phenotype could not be clearly established.

^d^ *avrLm4-avrLm7*, isolates virulent on both *Rlm4* and *Rlm7*; *avrLm4-AvrLm7*, isolates avirulent on *Rlm7* only; *AvrLm4-AvrLm7*, isolates avirulent on both *Rlm4* and *Rlm7;* nd*,* phenotypic interaction on *Rlm4* is missing.

**Supplementary Table S3. Southern blot analysis of mutational events in virulent alleles of *AvrLm7*.**

| Isolate ID ^a^ | PCR^b^ | *AvrLm4-7* sequence data | Size (kb) of hybridized bands after digestion with : | | |
| --- | --- | --- | --- | --- | --- |
|  |  |  | *Xba*I | *Hpa*I | *Spe*I |
| v23.1.3 | + | Wild type | 4.2^c^ | 1.9 and 0.5 | 3.3 |
| G06-E117 | - | - | no signal | no signal | no signal |
| G06-E205 | - | - | no signal | no signal | no signal |
| G07-C434 | - | - | no signal | no signal | no signal |
| G07-E1027 | - | - | no signal | no signal | no signal |
| G07-E1069 | - | - | no signal | no signal | no signal |
| G07-E492 | - | - | no signal | no signal | no signal |
| G07-C1009 | - | - | no signal | no signal | no signal |
| G08-C1222 | - | - | no signal | no signal | no signal |
| G08-E1028 | - | - | no signal | no signal | no signal |
| G08-E1321 | - | - | no signal | no signal | no signal |
| G08-E1167 | - | - | 1.9 (weak) | **1.9, 0.5 and 2.4 (weak)** | no signal |
| Nz-T4 | - | - | 1.9 (weak) | no signal | no signal |
| G07-E1026 | + | RIPped allele | **4.0 and 1.9** | 1.9 and 0.5 | **3.3, 2.8 and 0.5** |
| G07-E238 | + | RIPped allele | 1.9 | **1.9, 0.5 and 2.4** | 3.3 |
| G08-E1080 | + | RIPped allele | 1.9 | 1.9 and 0.5 | no signal |
| G07-C484 | + | RIPped allele with a few unresolved RIPped bases | 1.9 | 1.9 and 0.5 | **3.3, 2.8 and 0.5** |
| G08-C1052 | + | RIPped allele with a few unresolved RIPped bases | **4.2 and 1.9** | 1.9 and 0.5 | 3.3 |
| G08-E1474 | + | RIPped allele with a few unresolved RIPped bases | **4.2 and 1.9** | 1.9 and 0.5 | **3.3, 2.8 and 0.5** |

^a^ Nineteen virulent *Leptosphaeria maculans* field isolates were compared to the reference, avirulent isolate v23.1.3.

^b^ +, the *AvrLm4-7* gene can be amplified by PCR with both internal and external primers; -, *AvrLm4-7* cannot be amplified whatever the pair of primers used.

^c^ values in bold are indicative of the occurrence of two copies of *AvrLm4-7* with distinct RIP mutations affecting the sequence of the restriction site(s).

**Supplementary Table S4. Primers and PCR conditions used in this study.**

| Markers ^a^ | Primers (5'-3') | | | Hybridization temperature (°C) | Elongation time (s) |
| --- | --- | --- | --- | --- | --- |
|  | Forward (F) | Reverse (R) | |  |  |
| *AvrLm4-7* amplification and sequencing | |  | |  |  |
| AvrLm4-7ext-F & -R | TATCGCATACCAAACATTAGGC | GATGGATCAACCGCTAACAA | | 60 | 90 |
| AvrLm4-7Int-F & -R | ATATCTGGAGAAATTCGCTATC | CCAAGGGTCGGTAGTTATGC | | 60 | 30 |
| AvrLm4-7ext-F2 | ATTTGCTACACTAGATTATAC | - | | 60 | - |
| AvrLm4-7ext-F3 | AACCCTGCTAGATAGGTAAGCT | - | | 60 | - |
| High Resolution Melting PCR | |  | |  |  |
| AvrLm7-HRM | AACATGCCACTATCCCTC | ACCTCCGTATCTTTAGTC | | 54 | 45 |
| Quantitative PCR |  |  | |  |  |
| AVR47-QA-F & -R | GCCCTGCATAACTACCGAC | TCCTGGCCAAATATAACTCC | | 60 | 15 |
| Actine | AAGAGCGGTGATTTCCTTCT | AGTGCGATGTCGATGTCAG | | 60 | 15 |
| Minisatellite markers |  |  | |  |  |
| MinLm3 | GGCTCGGTCGGTTAGTTA | AATGATGTACAGGACGGGATTT | | 60 | 60 |
| MinLm6 | GGAAGGAACACACGGTGAC | | AATTGAATGATTTGCGACACA | 60 | 60 |
| MinLm8 | ATTTGCTGGCGGTGTAGGTA | TGTTTGTACATGTGGTAAGTAAAGCA | | 60 | 60 |
| MinLm1377 | CGTCCAGTCGTGCCTGCCTTTG | GGTGCATGTCTTGGCGGACCATT | | 58 | 60 |
| MinLm2451 | GGGGCGAATGGTATGTTTATAGT | CGGACACAATACTCACCACCTC | | 58 | 60 |
| Min_4_22 | GCAGAACGAGACCGAGTACA | | GTTGGGCCTGGTCATAGAGA | 60 | 60 |
| Min_6_59 | GAGAAACTTGTCTGGCCTGG | | GAGGCCAATGTCCAATGTGT | 60 | 60 |
| Tail PCR |  |  | |  |  |
| Tail-GD1 | CTCAAGGCACTTGTCCCAC | - | | 59 | - |
| Tail-GD2 | CCACAAAGACTGAAGCTTTGAATG | - | | 61 | - |
| Tail-GD3 | ATATGACGGCCAAAGTCCA | - | | 59 | - |
| AD1 ^b^ | - | NTCGA(G/C)T(A/T)T(G/C)G(A/T)GTT | | - | - |
| AD2 | - | NGTCGA(G/C)(A/T)GANA(A/T)GAA | | - | - |
| AD3 | - | (A/T)GTGNAG(A/T)ANCANAGA | | - | - |

^a^ For location of primers on the sequence, see Figure 3a.

^b^ Arbitrary Degenerated (AD) primers used in association with *AvrLm4-7* primers (Liu & Whittier, 1995)
